# Thrombopoietin signaling to chromatin elicits rapid and pervasive epigenome remodeling within poised chromatin architectures

**DOI:** 10.1101/gr.227272.117

**Published:** 2018-03

**Authors:** Federico Comoglio, Hyun Jung Park, Stefan Schoenfelder, Iros Barozzi, Daniel Bode, Peter Fraser, Anthony R. Green

**Affiliations:** 1 Cambridge Institute for Medical Research, Medical Research Council/Wellcome Trust Stem Cell Institute, and Department of Haematology, University of Cambridge, Cambridge CB2 0XY, United Kingdom;; 2 Nuclear Dynamics Programme, The Babraham Institute, Cambridge CB22 3AT, United Kingdom;; 3 Lawrence Berkeley National Laboratory, Berkeley, California 94720, USA;; 4 Department of Biological Science, Florida State University, Tallahassee, Florida 32301, USA;; 5 Department of Haematology, Addenbrooke's Hospital, Cambridge CB2 0XY, United Kingdom

## Abstract

Thrombopoietin (TPO) is a critical cytokine regulating hematopoietic stem cell maintenance and differentiation into the megakaryocytic lineage. However, the transcriptional and chromatin dynamics elicited by TPO signaling are poorly understood. Here, we study the immediate early transcriptional and *cis*-regulatory responses to TPO in hematopoietic stem/progenitor cells (HSPCs) and use this paradigm of cytokine signaling to chromatin to dissect the relationship between *cis*-regulatory activity and chromatin architecture. We show that TPO profoundly alters the transcriptome of HSPCs, with key hematopoietic regulators being transcriptionally repressed within 30 min of TPO. By examining *cis*-regulatory dynamics and chromatin architectures, we demonstrate that these changes are accompanied by rapid and extensive epigenome remodeling of *cis*-regulatory landscapes that is spatially coordinated within topologically associating domains (TADs). Moreover, TPO-responsive enhancers are spatially clustered and engage in preferential homotypic intra- and inter-TAD interactions that are largely refractory to TPO signaling. By further examining the link between *cis*-regulatory dynamics and chromatin looping, we show that rapid modulation of *cis*-regulatory activity is largely independent of chromatin looping dynamics. Finally, we show that, although activated and repressed *cis*-regulatory elements share remarkably similar DNA sequence compositions, transcription factor binding patterns accurately predict rapid *cis*-regulatory responses to TPO.

Hematopoiesis—the formation of blood cellular components—is an exquisitely characterized process in which the signaling consequences of extracellular stimuli, such as cytokines and growth factors, must be coherently integrated with chromatin structures to regulate cell-type–specific transcriptional programs (Rieger and Schroeder 2012). Specification of blood cell types is thought to proceed in a hierarchical fashion. At the apex of the hematopoietic hierarchy are multipotent hematopoietic stem cells (HSCs), which are able both to self-renew and to generate all blood cell lineages through differentiation into increasingly mature progenitor cells (Eaves 2015).

Lineage choice and commitment throughout hematopoiesis entails induction of lineage-specific gene regulatory networks and repression of lineage-inappropriate genes (Rieger and Schroeder 2012). These transcriptional programs are the result of coordinated waves of activation and decommissioning of unique constellations of *cis*-regulatory elements such as promoters and enhancers (Spitz and Furlong 2012). Cell-type–specific access to *cis*-regulatory information reflects the combinatorial activity of transcription factors (TFs), which recruit chromatin modifiers to establish active or repressive chromatin environments at regulatory elements within a *cis*-regulatory repertoire (Reiter et al. 2017).

Recent epigenomic advances have made it possible to map these repertoires genome-wide and across a wide range of cell types and organisms (The ENCODE Project Consortium 2012; Shlyueva et al. 2014). Putative enhancers can be defined as DNase I hypersensitive sites (DHSs) marked by monomethylated lysine 4 on histone H3 (H3K4me1), and their activity can be inferred based on the concomitant presence of histone H3 acetylation at lysine 27 (H3K27ac) often accompanied by detectable production of enhancer RNAs (eRNAs) (Heintzman et al. 2007, 2009; Creyghton et al. 2010; Calo and Wysocka 2013; Li et al. 2016). However, the targets of enhancers cannot be accurately predicted solely based on genomic proximity since transcriptional regulation occurs within the three-dimensional nuclear space (Bonev and Cavalli 2016; Dekker and Mirny 2016). Enhancers can exert their regulatory function on the expression of distally located target genes through three-dimensional chromatin looping that results in the juxtaposition of enhancer(s) and target promoter(s) (Bouwman and de Laat 2015; Bonev and Cavalli 2016). Experimental tethering of an enhancer to its target gene promoter has been shown to result in transcriptional activation of the *Hbb* locus, demonstrating that enhancer–promoter contacts can induce gene activation, even in the absence of a key transcriptional activator (Deng et al. 2012).

Chromosome conformation capture (3C)-based methods, particularly high-throughput 3C (Hi-C), have enabled targeted or genome-wide mapping of chromatin architectures (Denker and De Laat 2016). These technologies provided critical insights into key structural and functional components of three-dimensional chromatin organization such as (1) A/B compartments (Lieberman-Aiden et al. 2009), also referred to as compartment domains (Rao et al. 2017), which are closely associated with open and closed chromatin domains, respectively; (2) topologically associating domains (TADs) (Dixon et al. 2012; Nora et al. 2012; Sexton et al. 2012), also referred to as contact domains (Rao et al. 2017), chromosomal units that spatially constrain *cis*-regulatory interactions; and (3) CTCF loops, also referred to as insulated neighborhoods (Hnisz et al. 2016) or loop domains (Rao et al. 2017). Although these studies suggested a hierarchical domain organization, recent studies based on acute depletion of CTCF or cohesin, or inactivation of the cohesin-loading factor NIPBL, demonstrated that A/B compartments and TADs are not hierarchically organized but represent independent structural (and possibly functional) units of 3D genome organization (Nora et al. 2017; Rao et al. 2017; Schwarzer et al. 2017; Wutz et al. 2017).

Enhancer–promoter communication preferentially occurs within TADs (Zhan et al. 2017). However, the high complexity of Hi-C libraries makes it impractical to systematically map *cis*-regulatory interactions with high resolution and coverage. To overcome this limitation, we recently developed Promoter Capture Hi-C (PCHi-C), a sequence capture approach that selectively enriches Hi-C libraries for interactions involving more than 22,000 annotated mouse promoters, thus allowing global mapping of promoter interactions at restriction fragment level resolution (Schoenfelder et al. 2015).

Collectively, studies in different systems across multiple species using 3C-based methods and derivatives identified two types of enhancer–promoter interactions: loops formed de novo and preexisting loops (Montavon et al. 2011; Apostolou et al. 2013; Melo et al. 2013; Eijkelenboom et al. 2014; Ghavi-Helm et al. 2014; Cruz-Molina et al. 2017; Freire-Pritchett et al. 2017). Although de novo (also called instructive) interactions appear concomitant with changes in the target gene activity, preexisting (also called permissive) interactions are formed prior to gene activation and are thought to facilitate timely transcriptional induction (de Laat and Duboule 2013; Bouwman and de Laat 2015). However, the relationship between signal-dependent modulation of *cis*-regulatory activity and chromatin looping remains poorly understood for several reasons. First, poised chromatin architectures have been primarily studied in relation to gene activation, and little is known about signaling-dependent modulation of gene repression. Second, studies analyzing this relationship on developmental timescales lack the temporal resolution necessary to capture the early chromatin changes induced by signaling events. Third, physical interactions other than promoter–enhancer loops, such as enhancer–enhancer interactions, received comparatively little attention, albeit they likely represent an important layer of gene regulation in the three-dimensional nuclear space (Ing-Simmons et al. 2015).

The cytokine thrombopoietin (TPO) is a critical regulator of megakaryopoiesis. TPO regulates megakaryocyte and platelet production by activating its receptor, MPL (Bartley et al. 1994; de Sauvage et al. 1994; Lok et al. 1994), inducing multiple signaling pathways, including Janus kinase (JAK)/signal transducer and activator of transcription (STAT), mitogen-activated protein kinase, and phosphatidylinositol-3-kinase (De Graaf and Metcalf 2011). The physiological role of TPO has been extensively studied in mouse knockout (KO) models. TPO KO mice exhibit severe thrombocytopenia with 90% reduction in platelet counts, indicating that TPO is the major physiological regulator of megakaryocyte and platelet production in vivo (de Sauvage et al. 1996). Moreover, TPO and *Mpl* KO mouse models revealed that TPO signaling is also vital for hematopoietic stem cell maintenance and self-renewal (Kimura et al. 1998; Solar et al. 1998; Buza-Vidas et al. 2006; Qian et al. 2007; Yoshihara et al. 2007). However, these studies largely focused on TPO signaling at the level of cytoplasmic signaling pathways and/or cellular behavior. In contrast, the transcriptional consequences and *cis*-regulatory dynamics of TPO signaling to chromatin remain largely unknown.

## Results

### The immediate early transcriptional response to TPO signaling

To capture the immediate early transcriptional response to TPO, we performed subcellular RNA-seq (Bhatt et al. 2012) before and after 30-min TPO stimulation of HPC-7 cells, a cytokine-dependent and karyotypically normal multipotent hematopoietic stem/progenitor cell line (Pinto do Ó et al. 1998). HPC-7 cells self-renew in the presence of stem cell factor (SCF) and undergo megakaryocytic differentiation following TPO stimulation.

We isolated and profiled biochemically fractionated chromatin-associated, nucleoplasmic, and cytoplasmic transcripts in two biological replicates. Principal component analysis and hierarchical clustering indicate high reproducibility between replicates, with the subcellular compartment explaining most of the observed variation in gene expression (PC1, 72%) (Supplemental Fig. S1A,B). As expected, this is largely driven by the high intronic content of chromatin-associated RNA-seq libraries, which are enriched for unprocessed and incompletely spliced transcripts (Supplemental Fig. S1C). In addition, analysis of small nuclear and small cytoplasmic RNA gene loci revealed compartment-specific transcript localization, confirming the high enrichment of our RNA fractions (Supplemental Fig. S1D).

**Figure 1.**
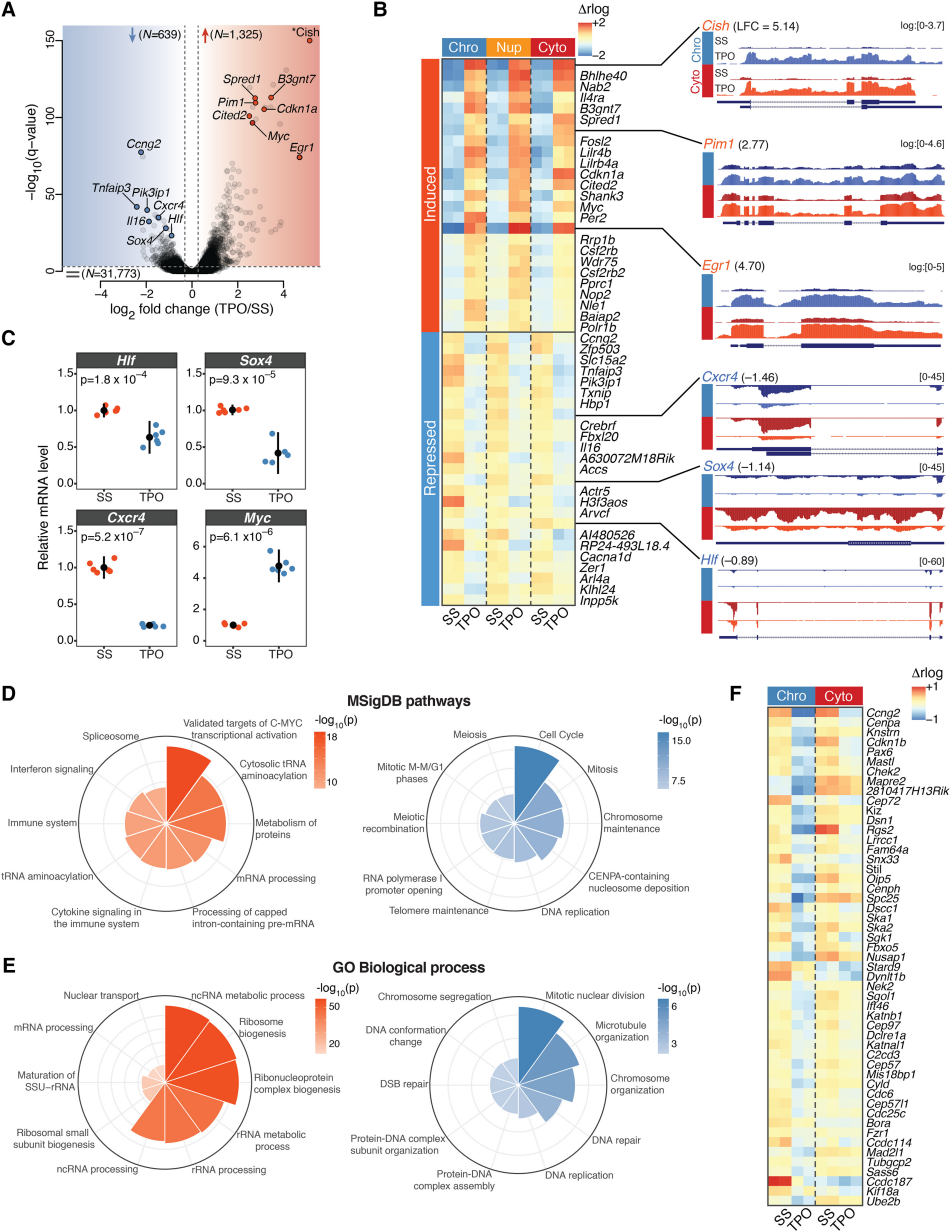
The immediate early transcriptional response to TPO signaling. (*A*) Volcano plot of gene transcription changes induced by 30 min stimulation of HPC-7 cells with TPO relative to serum-starved (SS) control cells, based on chromatin-associated RNA expression. Red and blue shaded regions enclose transcriptionally up- and down-regulated genes (*Q*-value <10^−3^), respectively. The total number of genes within each category is indicated. Representative hits are labeled. The asterisk denotes the most strongly induced gene (*Cish*, *Q* = 1.8 × 10^−241^), which was repositioned within the plot area. (*B*, *left*) Chromatin-associated (Chro), nucleoplasmic (Nup), and cytoplasmic (Cyto) RNA expression heatmap for the top 25 induced and top 25 repressed genes ranked by *Q*-value. Regularized log_2_ (rlog) expression values are row-mean subtracted. (*Right*) Representative tracks of differentially transcribed genes. Where indicated, RNA-seq coverage was log transformed with a pseudocount of 1. (LFC) log_2_ fold change. (*C*) mRNA expression levels of the indicated genes in TPO-treated (30 min) primary CD41^+^LSK cells, relative to serum-starved (SS) control cells, measured by quantitative RT-PCR. Error bars are mean ± SD (*n* = 6) from two mice. *P*-values are from a two-sided Welch's *t*-test. (*D*) Top 10 significantly enriched Molecular Signature Database (MSigDB) pathways for transcriptionally up- (*left*) and down-regulated (*right*) genes, ranked by binomial *P*-value. (*E*) Same as *D*, for Gene Ontology (GO) biological process terms. (*F*) Chromatin-associated (Chro) and cytoplasmic (Cyto) RNA expression heatmap of mitotic genes. Regularized log_2_ (rlog) expression values are row-mean subtracted.

We then analyzed the immediate early transcriptional consequences of TPO signaling at the chromatin level. We found that TPO transcriptionally up-regulated 1325 genes and repressed 639 genes (Fig. 1A). Importantly, significant changes in chromatin-associated RNA levels within 30 min of TPO stimulation only moderately overlapped changes detected in the cytoplasmic fraction (58% and 37% for up- and down-regulated genes, respectively) (Supplemental Fig. S1E) and exhibited significantly higher fold changes (Supplemental Fig. S1F). This suggests that RNA stability and post-transcriptional regulation contribute substantially to cytoplasmic gene expression changes even in response to transient signaling, underscoring the importance of profiling chromatin-associated or nascent transcripts when studying primary transcriptional responses. Unless otherwise stated, the remainder of our expression analyses focused on chromatin-associated transcripts.

The immediate early transcriptional consequences of TPO signaling to chromatin were highly divergent. On the one hand, genes most highly transcriptionally up-regulated by TPO included several targets of the canonical JAK/STAT signaling pathway (e.g., *Cish*, *Pim1*, *Cited2*, and *Egr1*) and the transcription factor MYC (Fig. 1A,B), suggesting broad housekeeping and survival functions. On the other hand, TPO led to the rapid transcriptional repression of key hematopoietic regulators such as *Hlf*, *Sox4*, and *Cxcr4* (Fig. 1B). Repression of these loci resulted in strongly decreased cytoplasmic RNA levels within 30 min of TPO, indicating that transcripts encoding these key regulators are subjected to rapid turnover. To validate our results in primary cells, we confirmed the rapid TPO-induced down-regulation of *Hlf*, *Sox4* and *Cxcr4*, as well as up-regulation of *Myc* by RT-qPCR in CD41^+^Lin^−^ Sca1^+^c-Kit^+^ (CD41^+^LSK) bone marrow cells (Fig. 1C; Nishikii et al. 2015), indicating that TPO elicits a common transcriptional program in megakaryocytic-biased hematopoietic progenitors.

To gain further insights into the nature of the transcriptional programs regulated by TPO, we subjected differentially transcribed genes to a Molecular Signatures Database (MSigDB) and Gene Ontology (GO) enrichment analyses. Transcriptionally up-regulated events were strongly enriched for housekeeping genes involved in RNA and protein metabolism, whose expression is largely driven by a MYC transcriptional program, and for genes that respond to cytokine signaling in the immune system (Fig. 1D,E). However, megakaryocytic-affiliated genes were not induced within 30 min of TPO (Supplemental Fig. S2), consistent with a slower induction kinetic (Park et al. 2015). In contrast, genes involved in mitosis, chromosome maintenance, and DNA repair were rapidly repressed by TPO (Fig. 1D–F). A distinctive feature of megakaryopoiesis is endomitosis, DNA replication in the absence of cell division that results from an incomplete M phase due to a failure in late cytokinesis (Bluteau et al. 2009). The rapid transcriptional repression of genes involved in mitotic nuclear division and microtubule organization (Fig. 1F) suggests that a cell cycle switch to endomitosis might occur very rapidly at the transcriptional level. Alternatively, it might represent a more general phenomenon of cell differentiation (Ruijtenberg and van den Heuvel 2016).

### TPO signaling elicits rapid and extensive epigenome remodeling of *cis*-regulatory landscapes

**Figure 2.**
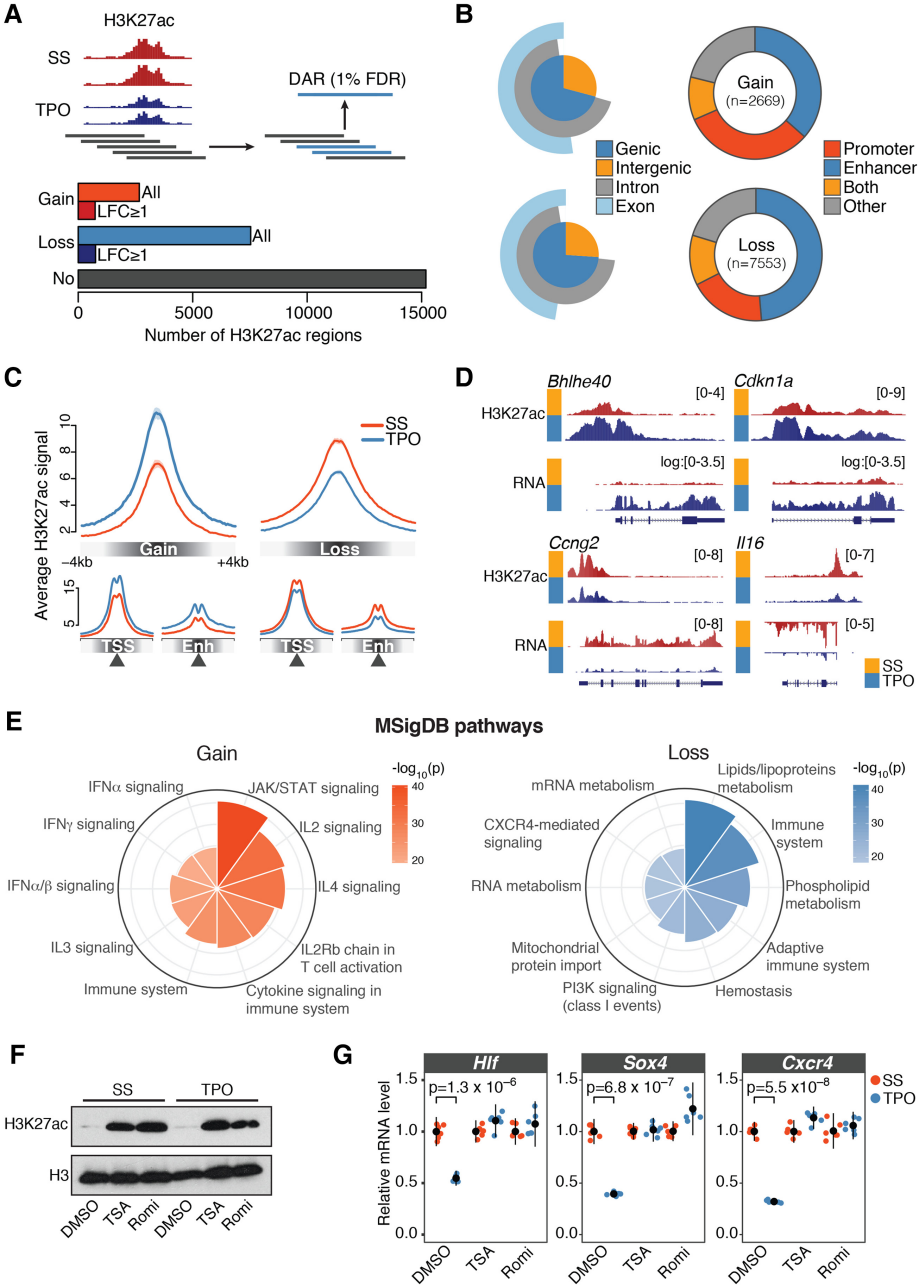
TPO signaling elicits rapid and extensive epigenome remodeling at *cis*-regulatory elements. (*A*, *top*) Schematic representation of the tiling-window approach used to identify differentially acetylated regions (DARs) from H3K27ac ChIP-seq profiles. Significant overlapping windows (blue rectangles) were merged to call DARs at 1% false discovery rate (FDR). (*Bottom*) Total number of activated (Gain) and repressed (Loss) DARs. H3K27ac regions not significantly altered by 30 min TPO (No) are also shown. (LFC) log_2_ fold change. (*B*, *left*) Annotation of DARs with respect to genomic compartments. (*Right*) Annotation of DARs with respect to *cis*-regulatory elements inferred from DNase-seq profiles in serum-starved HPC-7 cells (Methods). (*C*) Average normalized H3K27ac signal within ±4 kb of DAR summits (*top*), transcription start sites (TSSs), or enhancer summits (Enh). (*D*) Representative tracks of DARs. Where indicated, chromatin-associated RNA-seq coverage was log transformed with a pseudocount of 1. (*E*) Genomic regions enrichment of annotations tool (GREAT) analysis of DARs. Top 10 significantly enriched Molecular Signature Database (MSigDB) pathways for gene associated with DARs, ranked by binomial *P*-value. (*F*) Western blot analysis of H3K27ac and total H3 levels in HPC-7 cells pretreated for 30 min with DMSO, Trichostatin A (TSA), or Romidepsin (Romi), before and after 30 min TPO stimulation. (*G*) Corresponding mRNA expression levels of the indicated genes relative to serum-starved (SS) control cells, measured by quantitative RT-PCR. Error bars are mean ± SD (*n* = 6) from two biological replicates. *P*-values are from a two-sided Welch's *t*-test.

Next, we set out to investigate the chromatin dynamics underlying the immediate early response to TPO by surveying the activity of *cis*-regulatory elements before and after 30 min TPO stimulation of HPC-7 cells. To this end, we profiled H3K27ac genome-wide by chromatin immunoprecipitation sequencing (ChIP- seq) in two biological replicates and used a sliding window-based approach to detect genomic regions exhibiting significantly altered H3K27ac levels at 1% false discovery rate (FDR) (Fig. 2A; Lun and Smyth 2015; Methods).

Our analysis identified 10,222 differentially acetylated regions (DARs) exhibiting significantly increased (Gain, *n* = 2669) or decreased (Loss, *n* = 7553) H3K27ac levels within 30 min of TPO treatment (Fig. 2A). This number represents a sizeable fraction (40%) of genomic regions marked by H3K27ac in basal condition, demonstrating widespread (albeit not global) changes of *cis*-regulatory activity in response to transient cytokine signaling (Fig. 2A). Activated and repressed DARs showed overlapping size distributions with a median length of 3.4 kb (Supplemental Fig. S3A). However, the two sets differed in the magnitude of their response to TPO, with activated DARs exhibiting significantly stronger changes in H3K27ac levels (Fig. 2A; Supplemental Fig. S3B). We ruled out the possibility that these broad acetylation changes could stem from technical biases, because H3K27ac promoter signals at the 1000 most highly expressed genes unaffected by TPO were highly correlated (*r* = 0.94–0.99) and exhibited no systematic difference between replicates and conditions (Supplemental Fig. S3C). In addition, changes in the abundance of enhancer RNA (eRNA) transcripts at differentially acetylated intergenic enhancers correlated with changes in H3K27ac levels, further validating our DAR calls (Supplemental Fig. S3D). Moreover, to rule out the possibility that differential acetylation events reflect global changes in H3 occupancy, we measured H3K27ac and total H3 levels at a set of differentially acetylated loci by ChIP-qPCR (Supplemental Fig. S3E). This analysis indicates that H3K27ac changes at DARs represent bona fide acetylation dynamics.

We next mapped DARs onto genomic compartments. Less than 30% of DARs were intergenic, with the majority of acetylation changes overlapping annotated genes (Fig. 2B). To more precisely localize *cis*-regulatory elements within DARs, we mapped active promoters and enhancers at high resolution using DNase-seq profiles in self-renewing HPC-7 cells (Wilson et al. 2016; Methods). The vast majority (80%) of DARs encompassed elements (DHSs) from these two regulatory classes (Fig. 2B–D).

To investigate the functional significance of DARs, we performed a Genomic Regions Enrichment of Annotations Tool (GREAT) analysis by linking DARs to their putative target genes (McLean et al. 2010). As expected, activated DARs were strongly linked with genes involved in cytokine and JAK/STAT signaling (Fig. 2E). In contrast, repressed DARs were significantly associated to genes involved in lipid metabolism and in the regulation of the immune system, particularly of adaptive immunity (Fig. 2E), indicating that TPO signaling represses the *cis*-regulatory elements of lymphoid-affiliated genes.

Finally, to test whether deacetylation of *cis*-regulatory elements is required for transcriptional repression of key hematopoietic regulators (Fig. 1B,C), we pretreated HPC-7 cells with DMSO or the HDAC inhibitors Trichostatin A (TSA) or Romidepsin (Romi) for 30 min (Fig. 2F) and measured gene expression changes induced by 30-min TPO stimulation by RT-qPCR. We found that whereas *Hlf*, *Sox4*, and *Cxcr4* were significantly down-regulated within 30 min of TPO treatment in DMSO-treated cells, TSA or Romi fully abrogated this response (Fig. 2G).

Together, these results demonstrate that TPO signaling elicits rapid and extensive epigenome remodeling at *cis*-regulatory elements, and histone deacetylation is necessary for transcriptional repression of key hematopoietic regulators.

### *Cis*-regulatory dynamics induced by TPO signaling are spatially coordinated within topological domains

**Figure 3.**
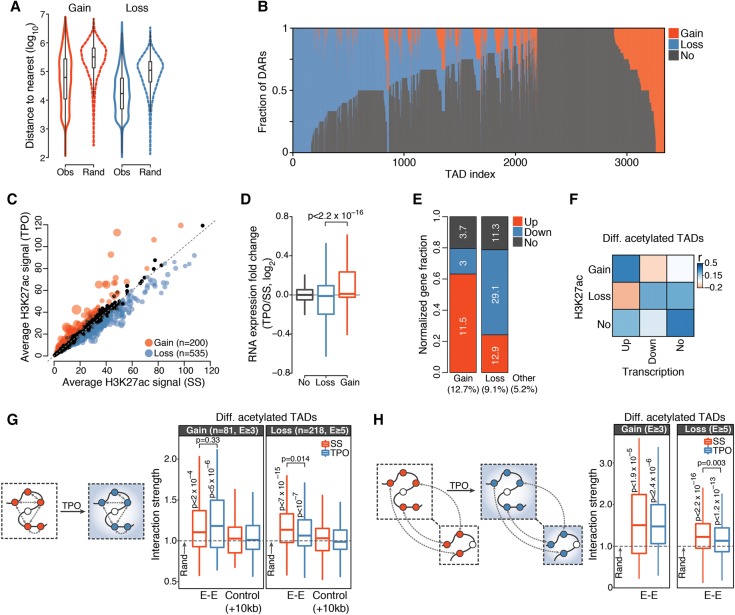
*Cis*-regulatory responses to TPO are spatially coordinated within the nucleus. (*A*) Distribution of observed (Obs) and expected (Rand) (Methods) genomic distances between nearest homotypic DARs. (*B*) Distribution of DARs within TADs detected in serum-starved HPC-7 cells and containing at least one H3K27ac region. Each TAD corresponds to a vertical line. (*C*) Average normalized H3K27ac signals per kilobase of TAD. Significantly differentially acetylated TADs are highlighted. Point sizes are proportional to −log_10_(*Q*-value). (*D*) Distribution of chromatin-associated RNA expression fold changes of genes localized within differentially acetylated TADs. *P*-value is from a Wilcoxon rank-sum test. (*E*) Relative fraction of differentially transcribed genes localized within differentially acetylated TADs, normalized by total number of genes within each category. The percentage of genes in each category is indicated inside the bar plot, whereas the percentage of differentially transcribed genes falling within each TAD class is indicated at the *bottom*. (*F*) Spearman's rank correlation matrix between frequency of DAR and frequency of differentially transcribed genes within differentially acetylated TADs. (*G*) Structured interaction matrix analysis (SIMA) of enhancer–enhancer interactions for differentially acetylated enhancers within differentially acetylated TADs. The interaction strength reflects the enrichment of Hi-C interactions relative to randomly sampled genomic regions. Interaction strength distributions for matched controls (Methods) are also shown. *P*-values are from a Wilcoxon signed rank test (for testing differences from random interactions) and from a Wilcoxon rank-sum test (for comparison between conditions). (*H*) Same as *G*, for enhancer–enhancer interactions between differentially acetylated TADs located within 20 Mb blocks.

We noted that homotypic DARs tended to strongly cluster along the chromatin fiber, suggesting spatial coordination within chromatin domains (Fig. 3A). Thus, we set out to systematically investigate how the rapid modulation of *cis*-regulatory activity induced by TPO is spatially coordinated within the nucleus. To this end, we generated Hi-C libraries from HPC-7 cells before and after 30-min TPO stimulation in two biological replicates and identified valid di-tags using the Hi-C Pro pipeline (Servant et al. 2015). We obtained a of total of 644 and 675 million valid di-tags in basal and TPO-treated conditions, respectively. Of these, 565 (87.7%) and 593 (87.9%) million valid di-tags, respectively, were intrachromosomal paired-end reads.

We first focused on megabase-sized chromatin domains. We found that higher-order chromatin structures, including A/B compartments, TAD boundary location and strength, and CTCF loops, were largely unaffected by transient TPO signaling (Supplemental Fig. S4A–F). This result is in line with previous studies of extracellular signaling that examined higher-order chromatin architectures 1 h after stimulation in other cellular systems (Jin et al. 2013; Le Dily et al. 2014).

We then analyzed the distribution of DARs within TADs identified in basal condition. This analysis revealed a clear spatial segregation of homotypic TPO-responsive DARs within topological domains (Fig. 3B), indicating that changes in *cis*-regulatory activity induced by TPO are spatially correlated at the level of TADs. To more formally investigate this aspect, we identified TADs exhibiting significantly altered H3K27ac levels in response to TPO at 1% FDR (Fig. 3C; Methods). This analysis singled out 200 (6% of all H3K27ac-marked TADs) globally induced and 535 (16%) globally repressed TADs (henceforth collectively called differentially acetylated TADs), allowing us to study topological domains that were mostly perturbed by TPO.

Compared to TADs with no significant changes in H3K27ac, differentially acetylated TADs were moderately enriched (approximately twofold) for differentially transcribed genes. As expected, genes in repressed TADs showed an overall lower expression than genes in activated TADs (Fig. 3D). Notably, although only 3% of down-regulated genes were located in activated TADs, a much higher fraction (12.9%) of up-regulated genes lay within repressed TADs (Fig. 3E). Thus, whereas down-regulated genes were virtually excluded from activated TADs, repressed TADs appeared at least partially permissive to transcriptional up-regulation (Fig. 3E).

Finally, we examined the relationship between transcriptional and *cis*-regulatory responses to TPO by correlating the number of differentially transcribed genes with the number of DARs located within the same differentially acetylated TAD. As expected, we found a marked correlation between increased *cis*-regulatory activity and transcriptional up-regulation (Fig. 3F). In contrast, we noticed a weaker association between repressed *cis*-regulatory elements and transcriptionally down-regulated genes (Fig. 3F). To further explore this aspect, we analyzed the distribution of differentially transcribed genes within all TADs exhibiting at least two homotypic DARs. To our surprise, we found that most TADs with repressed DARs did not show a corresponding detectable repression of genes located therein (Supplemental Fig. S4G), thus explaining the relatively weak association between *cis*-regulatory and transcriptional repression. Importantly, basal expression levels of these genes were significantly lower than for genes located within TADs hosting at least one differentially transcribed gene (Supplemental Fig. S4H). The lower expression levels reduce our ability to detect statistically significant further reductions in transcript levels. However, our results raise the possibility that repressed DARs within these loci might represent the first detectable step toward stable gene silencing of lineage-inappropriate genes.

### TPO-responsive enhancers are spatially clustered

At finer scales, physical interactions between promoter-distal sites appear to be widespread (Fullwood et al. 2009; Phillips-Cremins et al. 2013; Ghavi-Helm et al. 2014; Sahlén et al. 2015) and might function to provide specificity and robustness to enhancer–promoter interactions within *cis*-regulatory units (Markenscoff-Papadimitriou et al. 2014; Ing-Simmons et al. 2015). Previous work suggests that enhancer elements tend to cluster in the nuclear space in a cohesin-dependent manner (Ing-Simmons et al. 2015), but how enhancer–enhancer interactions are modulated by extracellular signaling remains largely unknown.

To investigate this aspect, we took advantage of the extensive and spatially compartmentalized epigenome remodeling induced by transient TPO signaling and analyzed enhancer–enhancer interactions within and between differentially acetylated TADs using a structured interaction matrix analysis (SIMA) (Lin et al. 2012). This method pools Hi-C interactions across a predefined set of genomic regions (in our case, enhancer elements) and computes their interaction strength relative to control regions randomly sampled from the same set of chromatin domains (differentially acetylated TADs). First, we focused on enhancer–enhancer interactions within activated and repressed TADs. In the absence of TPO, we found that homotypic enhancers interacted significantly more frequently with each other than expected based on a null model (Fig. 3G), indicating that they tend to congregate within topological domains. Importantly, this was not the case when enhancer coordinates were systematically shifted by 10 kb along the chromatin fiber, demonstrating that our analysis is well calibrated (Fig. 3G). We then examined the consequences of TPO signaling. Enhancer–enhancer interactions were only moderately perturbed within 30 min of TPO (Fig. 3G). Indeed, homotypic enhancers remained significantly clustered within differentially acetylated TADs, suggesting that TPO selectively modulates enhancer–enhancer interactions rather than altering them at a global scale.

Although by definition intra-TAD interactions occur more frequently than interactions spanning TAD boundaries, topological domains represent a modest twofold enrichment in interaction frequency (Dixon et al. 2012; Nora et al. 2012; Sexton et al. 2012). Therefore, we tested whether enhancers located within neighboring TPO-regulated TADs show evidence of spatial clustering (Methods). We found that, similarly to intra-TAD interactions, inter-TAD enhancer–enhancer interactions were significantly enriched over random expectation and only moderately perturbed by transient TPO signaling (Fig. 3H). This enrichment was further confirmed by an analysis of enhancer–enhancer interactions between CTCF loops (Supplemental Fig. S3F).

Together, these results indicate that TPO-responsive enhancers engage in preferential long-range intra- and inter-TAD interactions resulting in their clustering in the three-dimensional nuclear space, and TPO-induced changes in enhancer activity are at least partly uncoupled from this spatial clustering of enhancer elements.

### TPO-responsive super-enhancers control the expression of key hematopoietic regulators

Transcriptional enhancers not only interact with each other through long-range interactions but also within dense enhancer hotspots known as super-enhancers (SEs) or stretch-enhancers (Parker et al. 2013; Whyte et al. 2013; Ing-Simmons et al. 2015), which have been implicated in the transcriptional control of cell identity genes (Hnisz et al. 2013). Intriguingly, a recent study revealed that SE-associated genes are enriched for functional categories relevant to cytokine biology in mouse T lymphocytes (Vahedi et al. 2015). Therefore, we reasoned that SEs might be prime candidates for the integration of TPO signaling to chromatin.

**Figure 4.**
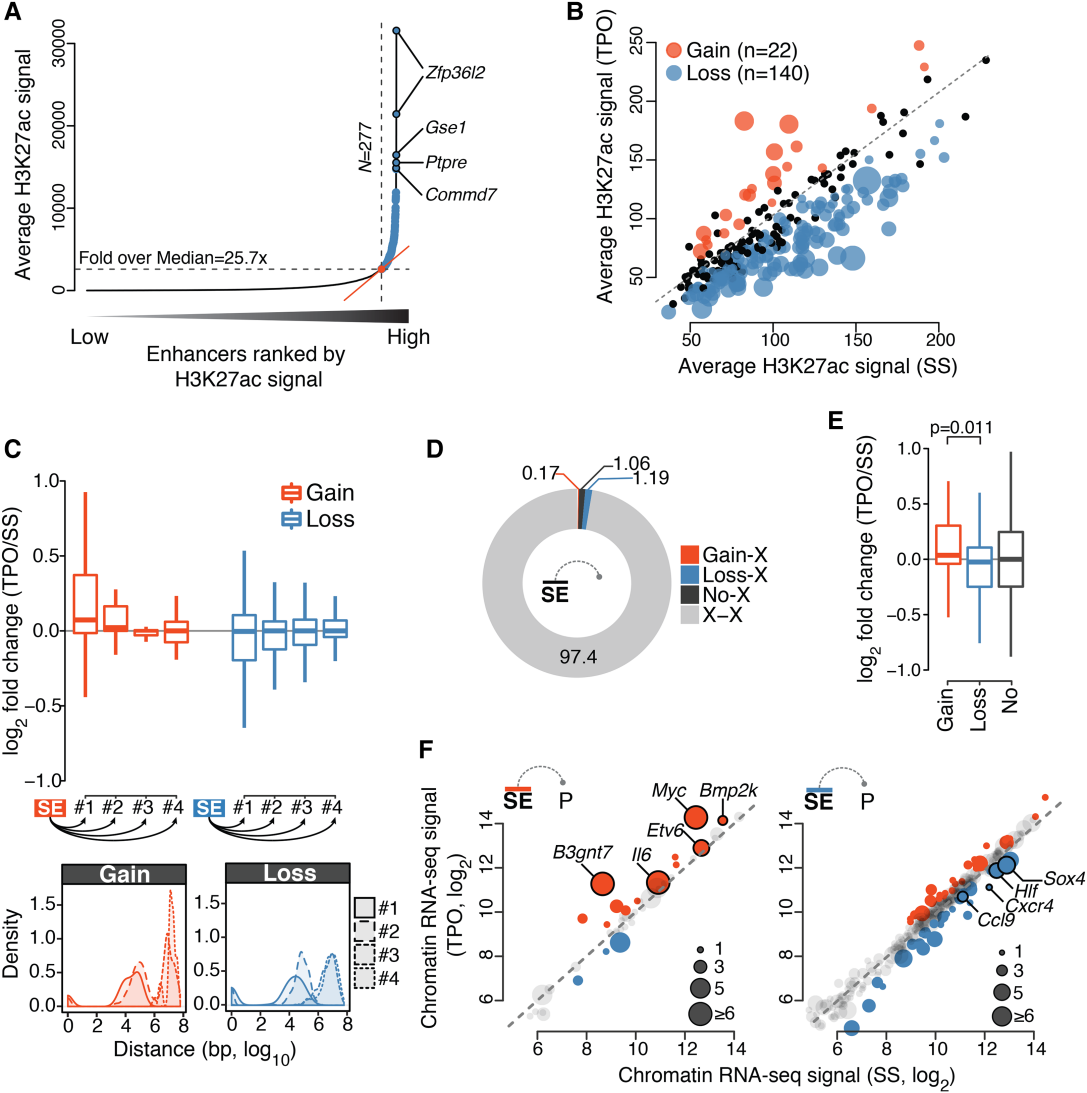
TPO signaling is integrated at super-enhancers. (*A*) Distribution of average (control-subtracted) normalized H3K27ac ChIP-seq signals at enhancer regions in serum starved HPC-7 cells. Super-enhancers (SEs, blue) exhibit exceptionally high H3K27ac levels. (*B*) Average normalized H3K27ac signal per kilobase of SE. Significantly differentially acetylated SEs are highlighted. Point sizes are proportional to −log_10_(*Q*-value). (*C*, *top*) Distribution of chromatin-associated RNA expression fold change for first, second, third, and fourth nearest gene to a differentially acetylated SE. (*Bottom*) Corresponding distribution of genomic distances. (*D*) Percentage of significant promoter Capture Hi-C interactions anchored at SEs. (X) any HindIII restriction fragment located outside SEs. (*E*) Distribution of chromatin-associated RNA expression fold changes of SE-target genes defined by promoter Capture Hi-C. *P*-value is from a Wilcoxon rank-sum test. (*F*) Chromatin-associated RNA expression (regularized log_2_ values) of gene targets of differentially acetylated SE as defined in *E*. Point sizes are proportional to the number of constituent enhancers exhibiting significant interactions with the gene promoter. Selected SE targets are labeled.

To test this hypothesis, we identified SEs in serum-starved HPC-7 cells (Fig. 4A; Methods) and analyzed their response to 30-min TPO stimulation. TPO signaling significantly perturbed the activity of more than half of all SEs (162 of 277, 58%) identified in basal condition. The vast majority of these TPO-responsive SEs (140 of 162, 86%) were significantly deacetylated within 30 min of TPO, indicating that TPO signaling primarily represses SE activity (Fig. 4B; Supplemental Fig. S5A,B). We next examined the consequences of this perturbation on the transcription of putative target genes as a function of their genomic distance to SEs. Differential acetylation of SEs was more frequently linked to transcriptional perturbation of SE-proximal genes located within genomic distances <1 Mb (Fig. 4C; Supplemental Fig. S5C), with 27% of activated SEs and 22% of repressed SEs localizing within the same CTCF loop as their closest differentially transcribed gene. Nevertheless, the majority of genes located in close proximity to TPO-responsive SEs exhibited little or no detectable transcriptional changes within 30 min of TPO (Fig. 4C). This result suggests that SE chromatin dynamics might often precede transcriptional changes at target genes or that genomic proximity is not an accurate predictor of SE targets.

To more accurately link SEs to their target genes, we enriched our Hi-C libraries for interactions anchored at 22,225 annotated gene promoters within the mouse genome using Promoter Capture Hi-C (PCHi-C) (Schoenfelder et al. 2015), for a total of 445 and 407 million intrachromosomal valid di-tags in basal and TPO-treated conditions, respectively. Data normalization and interaction detection from two biological replicates using the CHiCAGO pipeline (Cairns et al. 2016) resulted in 192,634 and 181,235 statistically significant (CHiCAGO score ≥5) promoter-anchored long-range *cis*-interactions for basal and TPO-treated conditions, respectively. Focusing on SEs, we found that approximately 2.4% of all significant PCHi-C interactions linked promoters to SE constituents (Fig. 4D), allowing us to infer the targets of differentially acetylated SEs based on spatial proximity. Although most SE-interacting genes showed no detectable transcriptional changes within 30 min of TPO, we found that targets of induced and repressed SEs were enriched for transcriptionally up- and down-regulated genes, respectively, and these included known SE targets such as *Myc* and *Etv6* (Fig. 4E,F; Khan and Zhang 2016). Promoters of key hematopoietic regulators rapidly repressed by TPO signaling (*Hlf*, *Sox4*, and *Cxcr4*) (Fig. 1B,C) were highly connected to constituent enhancers within repressed SEs (Fig. 4F), suggesting that their expression is controlled from a distance through TPO-responsive SEs.

### Rapid modulation of *cis*-regulatory activity is largely independent of chromatin looping dynamics

**Figure 5.**
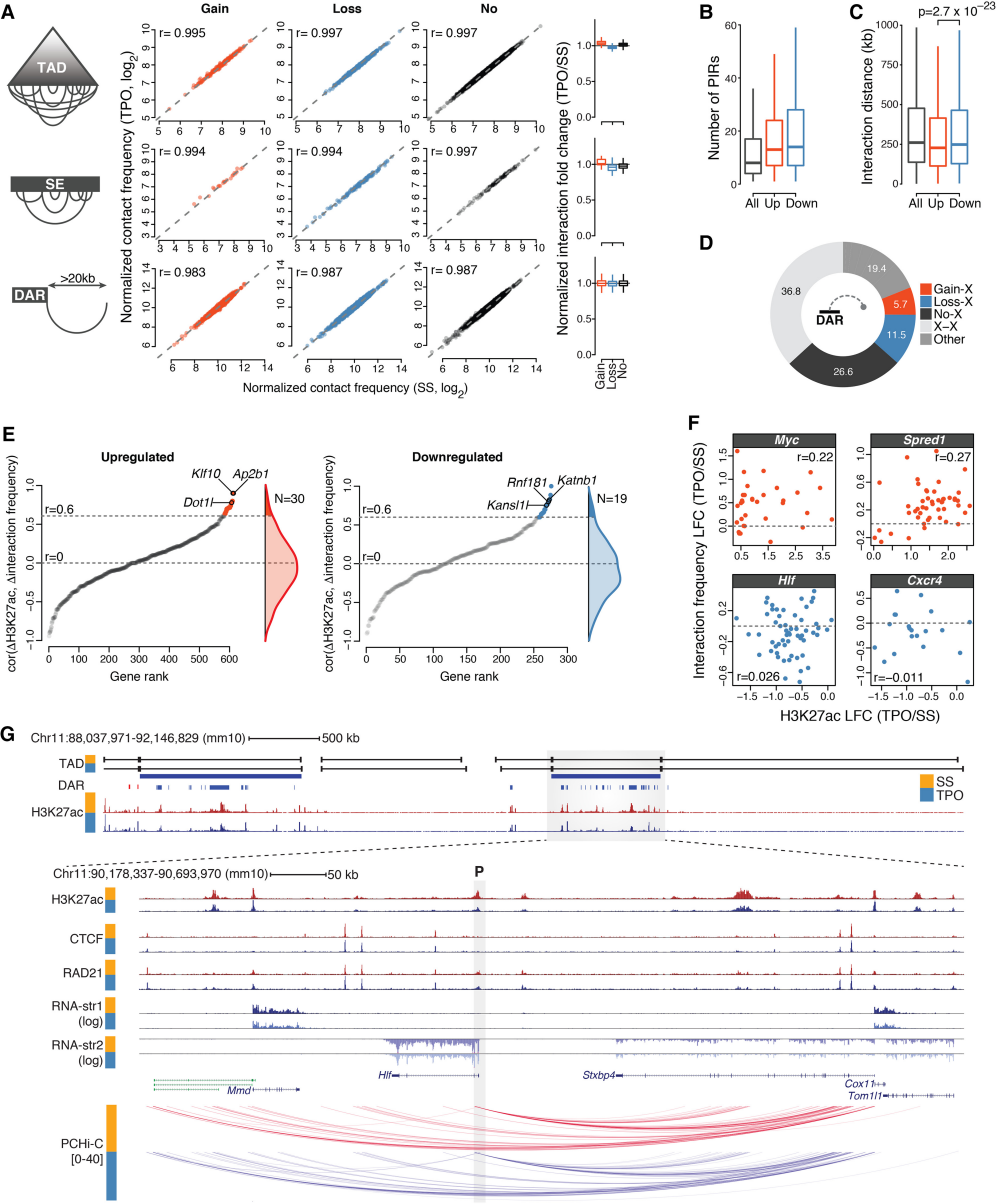
Rapid modulation of *cis*-regulatory activities within poised chromatin architectures. (*A*) Normalized Hi-C contact frequencies for intra-TAD and intra-SE interactions (per kilobase of element), and for interactions anchored at DARs spanning more than 20 kb. Spearman's rank correlation coefficients (*r*) are shown. Box plots (*right*) summarize normalized interaction fold change distributions. (*B*) Distribution of the number of promoter-interacting regions (PIRs) for all baited promoters (All) and promoters of transcriptionally up- and down-regulated genes. (*C*) Distribution of interaction distances for all baited promoters (All) and promoters of transcriptionally up- and down-regulated genes. *P*-value is from a Wilcoxon rank-sum test. (*D*) Percentage of significant promoter Capture Hi-C interactions anchored at DARs. (X) any HindIII restriction fragment located outside DARs. (*E*) Relationship between *cis*-regulatory activity and chromatin architectures at *cis*-regulatory units for transcriptionally up- and down-regulated genes (Methods). Genes are ranked based on the Spearman's rank correlation coefficient (*r*) between normalized H3K27ac fold change (TPO/SS) and normalized Capture Hi-C interaction fold change at target DARs. Only promoters exhibiting significant interactions with at least five distinct DAR-containing HindIII restriction fragments were considered. Genes exhibiting significant correlations are colored, and representative hits are labeled. (*F*) Representative examples from the analysis in *E*. Each dot corresponds to an HindIII restriction fragment. (LFC) log_2_ fold change. (*G*) Epigenomic configuration of the *Hlf* locus. Statistically significant promoter Capture Hi-C interactions within the *Hlf* TAD are shown. The gray shaded rectangle denotes the position of the baited *Hlf* promoter (P). (str) strand.

Next, we tested whether the pervasive epigenome remodeling observed within differentially acetylated TADs and within SEs was accompanied by systematic changes in chromatin looping. To this end, we computed normalized Hi-C contact frequencies within these chromatin domains before and after 30-min TPO stimulation. Our analysis revealed no systematic differences in contact frequencies between conditions, which exhibited nearly perfectly correlated Hi-C signals (*r* > 0.99) (Fig. 5A). This was further confirmed by a systematic analysis of chromatin interactions anchored at DARs (Fig. 5A). These results indicate that, despite substantial changes in both transcriptome and epigenome, chromatin architectures at differentially acetylated TADs and SEs were essentially unaltered following TPO stimulation.

We then focused on the spatial chromatin architecture at differentially transcribed gene loci, taking advantage of the increased resolution offered by PCHi-C over Hi-C data. We found that most promoters of differentially transcribed genes engaged more than 10 promoter-interacting regions (PIRs) (with a median of 13 and 14 PIRs for transcriptionally up- and down-regulated genes, respectively) (Fig. 5B); although for transcriptionally up-regulated genes these interactions tended to span significantly shorter genomic distances (Fig. 5C), no major differences were found in the connectivity of these loci. In addition, 63% of all promoter interactions detected by PCHi-C were anchored at H3K27ac-marked regions (Fig. 5D), with 30.7% encompassing at least one DAR, indicating extensive coverage of these genomic regions.

These results prompted us to systematically correlate changes in H3K27ac levels with changes in PCHi-C interaction frequency within individual differentially transcribed *cis*-regulatory units (Methods). For each promoter, we defined a *cis*-regulatory unit as the union of all its H3K27ac-marked PIRs, and we set a threshold of at least five such regions being required to ensure robust estimates of H3K27ac fold changes and correlation coefficients. This resulted in a set of 907 differentially transcribed loci (Fig. 5E). We found that the vast majority of *cis*-regulatory units exhibited little or no correlation between changes in *cis*-regulatory activity and chromatin looping (Fig. 5E).

This result was further supported by a permutation test in which the connectivity of *cis*-regulatory units was randomly scrambled to derive a null distribution for the correlation coefficients (Methods). Our data revealed that only 5.4% of differentially transcribed gene loci being tested (49 of 907, corresponding to 4.8% and 6.7% of analyzed transcriptionally up- and down-regulated genes) exhibited a statistically significant correlation between acetylation and looping dynamics in response to transient TPO signaling (Fig. 5E). Neither rapidly induced genes (e.g., *Myc* and *Spred1*) (Fig. 1A) nor repressed loci encoding key hematopoietic regulators belonged to this set (Fig. 5F). Indeed, although these loci exhibited extensive differential acetylation within 30 min of TPO, this was not accompanied by a rewiring of chromatin loops (Fig. 5F,G; Supplemental Fig. S6).

Together, these results demonstrate that the extensive and spatially compartmentalized epigenome remodeling induced by transient TPO signaling takes place within poised chromatin architectures and suggest that the rapid cytokine-dependent modulation of *cis*-regulatory activity is largely independent of chromatin looping dynamics.

### TF binding patterns but not DNA sequence features accurately predict rapid *cis*-regulatory responses to TPO

**Figure 6.**
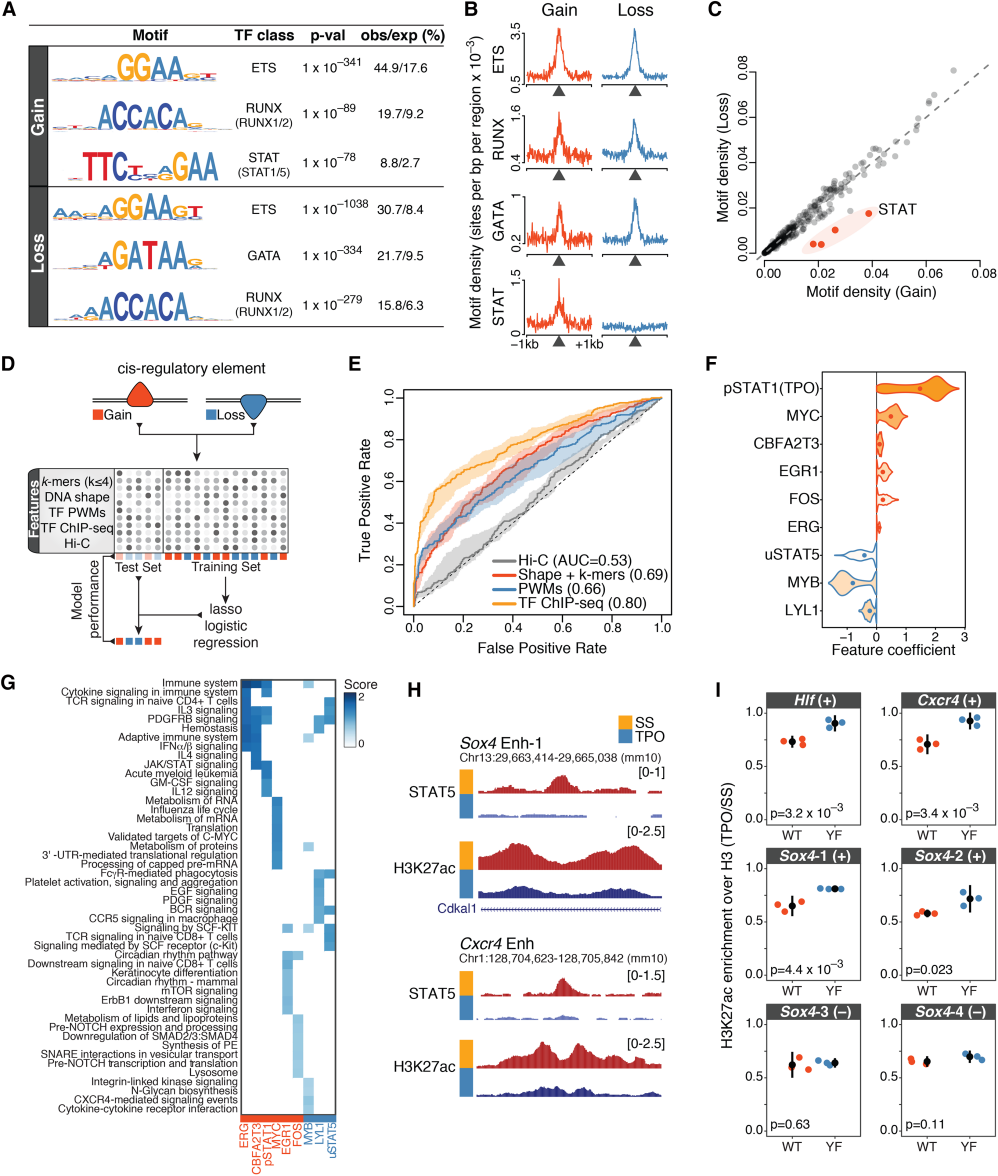
TF binding patterns accurately predict rapid *cis*-regulatory responses to TPO. (*A*) Top transcription factor motifs (position weight matrices [PWMs]) identified by de novo motif discovery analysis within 200 nt of the summit of differentially acetylated DHSs (Methods). The percentage of observed and expected motif occurrence is indicated. (*B*) Motif density (PWMs) for the indicated transcription factor motifs within ±1 kb of the summit of differentially acetylated DHSs. (*C*) Total motif density within 200 nt of the summit of differentially acetylated DHSs, for a collection of 363 vertebrate transcription factor PWMs. (*D*) Schematic outline of the statistical learning strategy used to predict how *cis*-regulatory elements within DARs respond to TPO. (*E*) Test set receiver operating characteristic (ROC) curve and area under the ROC curve (AUC) values for lasso models trained on the indicated sets of features. The shaded area is delimited by the ROC curves for models with highest and lowest AUC values, whereas the ROC curve for the model closest to mean AUC is shown. (*F*) Top-ranked features selected by bootstrap-Lasso for the model trained on TF ChIP-seq profiles, ranked by selection probability (stability). Violin plot lines are color-coded according to coefficient signs: (red) positive; (blue) negative. (*G*) Genomic regions enrichment of annotations tool (GREAT) analysis of differentially acetylated *cis*-regulatory elements bound by TFs in *F*. The top eight significantly enriched Molecular Signature Database (MSigDB) pathways were considered for each transcription factor, ranked by binomial *P*-value. (*H*) Representative tracks illustrating STAT5 binding dynamics at differentially acetylated enhancers within key hematopoietic gene loci. (*I*) Relative H3K27ac enrichment (TPO/SS, normalized to total H3 levels) at the indicated enhancer elements (uSTAT5-bound [+] or uSTAT5 negative [–, control]) for HPC-7 cells expressing wild-type (WT) or mutant (Y699F) STAT5B. Tested loci include the enhancers shown in *H*. Error bars are mean ± SD (*n* = 3). *P*-values are from a two-sided Welch's *t*-test.

Finally, we asked which TFs orchestrate the rapid *cis*-regulatory dynamics induced by TPO signaling. To identify TFs that control activated and repressed *cis*-regulatory elements within DARs, we subjected differentially acetylated DHSs to a de novo motif discovery analysis. We found that both activated and repressed DARs were strongly enriched for ETS motifs, which feature a central 5′-GGAA-3′ core, and for motifs recognized by the RUNX and GATA families of TFs (Fig. 6A,B). These motifs exhibited similar enrichments and overlapping distributions at activated and repressed DARs (Fig. 6B). In stark contrast, an interferon γ-activation sequence (GAS) motif, which is recognized by tyrosine-phosphorylated STAT (pSTAT) TFs, was specifically overrepresented at activated but not at repressed DARs (Fig. 6B), indicating that canonical JAK/STAT signaling is exclusively associated with DAR activation. Surprisingly, however, a systematic motif enrichment analysis based on 363 position weight matrices (PWMs) for 322 vertebrate TFs revealed that no TF motif other than those recognized by pSTATs exhibited a comparably specific association with either DAR class (Fig. 6C). Thus, despite exhibiting opposite response to TPO, activated and repressed *cis*-regulatory elements were characterized by a remarkably similar TF motif composition.

This result led us to perform an unbiased survey of DNA and chromatin features that might be able to predict rapid *cis*-regulatory responses to TPO signaling. To this end, we considered activated and repressed *cis*-regulatory elements and trained least absolute shrinkage and selection operator (lasso) logistic regression models (Tibshirani 1996) using different sets of features (Fig. 6D; Methods). These included DNA sequence *k*-mers (2 ≤ *k* ≤ 4), DNA shape features (Chiu et al. 2016), a collection of more than 1700 single and composite TF motifs (Diaferia et al. 2016) that was filtered for TFs expressed in HPC-7 cells, a collection of 29 ChIP-seq binding profiles for hematopoietic and other sequence-specific TFs in HPC-7 cells before (*n* = 26) and after (*n* = 3) 30-min TPO stimulation (generated as part of this study or previously published) (Wilson et al. 2010, 2016; Park et al. 2015; Supplemental Table S1), and normalized Hi-C signals as a proxy for interaction frequencies. Model performances were evaluated on a test set consisting of differentially acetylated *cis*-regulatory elements that were not used for learning model parameters (Fig. 6D).

Although DNA sequence features were only moderately predictive for *cis*-regulatory responses to TPO (area under the receiver operating characteristic curve, AUC <0.70), in vivo TF binding profiles were able to accurately predict changes in *cis*-regulatory activity (AUC = 0.80) (Fig. 6E). In contrast, Hi-C signals showed virtually no predictive power (AUC = 0.53) (Fig. 6E), further supporting the notion that these changes occur within poised chromatin architectures. In addition, more complex models combining sequence and chromatin features did not outperform models learned on TF binding profiles alone (Supplemental Fig. S7A), suggesting that key TFs were already included within our chromatin feature set. Furthermore, these results were validated by an independent learning scheme using random forest classifiers (Supplemental Fig. S7B; Breiman 2001).

Next, we focused on the models learned on TF binding profiles and sought to identify the chromatin features that contribute the most to model predictions by estimating feature stability coefficients using bootstrap-Lasso (Comoglio and Paro 2014; Comoglio et al. 2015). Intuitively, the more a feature is necessary for accurate predictions, the higher its stability value. Our analysis revealed that preexisting MYC binding and binding of pSTAT1 upon TPO stimulation were stably associated with the activation of *cis*-regulatory elements (Fig. 6F; Supplemental Fig. S7C). In contrast, binding of MYB, LYL1, and tyrosine unphosphorylated STAT5 (uSTAT5) in the absence of TPO was predictive for repression of *cis*-regulatory elements (Fig. 6F; Supplemental Fig. S7C). Notably, DARs bound by stably selected features were significantly associated with distinct signaling pathways and metabolic functions, spanning the whole spectrum of biological processes regulated by TPO (Fig. 6G).

We previously demonstrated that in the absence of TPO, chromatin-bound uSTAT5 restrains a megakaryocytic transcriptional program in hematopoietic stem/progenitor cells, and TPO-mediated phosphorylation of STAT5 triggers a genome-wide relocation of STAT5 to canonical GAS motifs (Park et al. 2015). Intriguingly, uSTAT5 knockdown not only up-regulated megakaryocytic-affiliated genes, but it also repressed lineage-inappropriate genes including lymphoid- and pregranulocyte/monocyte-affiliated genes (Park et al. 2015). This result, along with our feature importance analysis (Fig. 6F), led us to hypothesize that uSTAT5 binding might be required to maintain the activity of *cis*-regulatory elements within gene loci rapidly repressed by TPO. To test this hypothesis, we took advantage of a mutant (Y699F) STAT5B that ablates TPO-induced tyrosine phosphorylation of STAT5 and does not act as a dominant negative (Park et al. 2015). We expressed wild-type or mutant STAT5B in HPC-7 cells and measured H3K27ac levels at uSTAT5-bound enhancers within the *Hlf*, *Sox4*, and *Cxcr4* loci (Fig. 6H) by ChIP-qPCR before and after 30-min TPO stimulation. The Y699F mutant significantly attenuated deacetylation at uSTAT5-bound enhancers in response to TPO compared to wild type (Fig. 6I), indicating that uSTAT5 binding is required to maintain the activity of these elements.

## Discussion

In this study, we focused on the immediate early consequences of TPO signaling to chromatin and examined the relationship between signal-dependent modulation of *cis*-regulatory activity and chromatin looping. Our work provides three key findings.

First, we present evidence for rapid and pervasive changes in *cis*-regulatory activity that are readily detectable within 30 min of TPO. Interestingly, this widespread epigenome remodeling encompasses up to 40% of active genomic regions marked by H3K27ac prior to TPO stimulation, indicating that short-term cytokine signaling is sufficient to profoundly alter chromatin states at *cis*-regulatory elements. Notably, a sizeable fraction of these remodeling events corresponds to histone deacetylation of enhancer elements associated with genes playing key roles in innate and adaptive immune cells. This rapid deacetylation of enhancer elements likely represents the first step toward decommissioning of active enhancers associated with lineage-inappropriate transcriptional programs (Smith and Shilatifard 2014) and suggests that TPO signaling rapidly dismantles *cis*-regulatory networks affiliated to alternative lineages.

Second, we demonstrate that TPO-induced modulation of *cis*-regulatory units occur largely independently of alterations in chromatin looping, indicating that a responsiveness to TPO is hardwired into a poised HSPC spatial genome architecture prior to cytokine exposure. It is important to note, however, that TPO-dependent modulation of short-range interactions between enhancers and promoters would not be detectable in our PCHi-C data.

A previous Hi-C analysis of chromatin structures after 1-h TNF stimulation of primary human fibroblast cells showed that TNF-responsive enhancers are already in contact with their target promoters prior to stimulation, and these preexisting chromatin structures appear to be strong predictor of gene induction (Jin et al. 2013). Similarly, preformed chromatin loops have also been detected at inducible gene loci regulated by TP53, FOXO3, and hormone receptor binding by 3C or circular chromosome conformation capture (4C) sequencing (Jin et al. 2013; Melo et al. 2013; Eijkelenboom et al. 2014). Our results indicate that poised chromatin architectures not only provide permissive regulatory topologies for transcriptional induction, but also a platform for rapid signaling-dependent repression of *cis*-regulatory units. Therefore, it is plausible that disruption of preformed chromatin architectures prior to TPO stimulation might similarly impair both activation and repression of *cis*-regulatory units in response to TPO signaling. However, further work will be required to formally test this hypothesis.

Third, we show that TPO signaling can induce opposite responses at *cis*-regulatory elements exhibiting markedly similar DNA sequence compositions and regulatory codes. This unexpected finding raises the question about which molecular determinants direct these opposing events. Regulatory instructions are encoded in DNA sequences by the identity, frequency, affinity, and grammar of TF binding sites. However, how these parameters dictate *cis*-regulatory dynamics is poorly understood (Spitz and Furlong 2012; Levo and Segal 2014). Our results indicate that, with the notable exception of pSTATs, the mere presence or absence of binding motifs for specific TFs cannot predict individual *cis*-regulatory responses to TPO and raise the possibility that orientation and spacing of TF motifs might play a role in determining these responses. Consistent with this concept, a recent work demonstrated that a different motif grammar based on the same building blocks defines activating and repressing *cis*-regulatory elements in rod photoreceptors (White et al. 2016). Moreover, several studies showed that a subset of TFs can act both as repressor or activator depending on cellular states, sequence contexts, and binding of cofactors (Méthot and Basler 1999; Sharrocks 2001; Nayak et al. 2009; Hollenhorst et al. 2011; Liu et al. 2014; Sánchez-Tilló et al. 2015). Deciphering the motif grammar that distinguishes activated and repressed *cis*-regulatory elements should be an important goal of future studies. To this end, statistical learning models considering motif orientation and spacing could be used to guide the design of massively parallel reporter assays based on synthetic regulatory elements or more targeted in vitro assays (Inoue and Ahituv 2015; Fiore and Cohen 2016).

Although our analysis suggests that regulatory priming in multipotent progenitors does not appear to be constrained by sequence composition, in vivo TF binding patterns are able to accurately predict rapid *cis*-regulatory responses to TPO. Interestingly, feature importance analysis indicates that preexisting TF binding prior to TPO stimulation significantly contributes to these predictions. This notion is further supported by the targeted experimental analysis of uSTAT5-bound enhancers, where preexisting uSTAT5 binding is predictive for the repression of these regulatory elements. Moreover, our statistical models identified other potential key players mediating rapid *cis*-regulatory responses to TPO such as MYB and LYL1. These hematopoietic TFs are critically involved in the maintenance of HSPCs as well as in lymphoid differentiation (Capron et al. 2006; Fahl et al. 2009; Lieu and Reddy 2009; Souroullas et al. 2009; Greig et al. 2010). Our results suggest that MYB and LYL1 binding prior to TPO stimulation predicts deacetylation of *cis*-regulatory elements associated with the lymphoid lineage. Therefore, it is tempting to speculate that TPO supports megakaryocytic lineage specification in part by restricting the developmental potential of multipotent progenitor cells. To this end, it would be interesting to further investigate whether TPO directly regulates chromatin binding and/or activity of MYB and LYL1 in HSPCs.

Taken together, our study unravels the multifaceted immediate early consequences of TPO signaling to chromatin and provides a paradigm for the integrative epigenomic analysis of cytokine signaling.

## Methods

### Cells and cell culture

HPC-7 cells were grown at 37°C and 5% CO_2_ in IMDM (Invitrogen) supplemented with 10% fetal calf serum, 10% SCF conditioned media (produced by the BHK/MKL cell line), 1% L-Glutamine, 1% penicillin/streptomycin, and 74.8 µM monothioglycerol (Sigma). Cell density was maintained between 5 × 10^5^ and 2 × 10^6^ cells/mL.

### Mice

Eight- to twelve-week-old C57BL/6 mice (Charles River) were used in this study. All mouse procedures were approved by the UK Home Office and the University of Cambridge Animal Welfare and Ethical Review Board.

### Serum starvation and cytokine stimulation

Cells were spun down at 1000 rpm for 5 min and washed once in PBS. The cell pellet was resuspended at a density of 1 × 10^6^ cells/mL in StemSpan SFEM medium (StemCell Technologies) and incubated for 4 h. Serum-starved cells were then stimulated with recombinant murine thrombopoietin (TPO, Peprotech) at 100 ng/mL for 30 min. TPO was diluted in a small volume of fresh StemSpan SFEM medium prior to stimulation.

### Isolation of CD41^+^LSK cells

Freshly isolated mouse bone marrow cells were stained with AF700-conjugated lineage cocktail (133313), APC-Cy7-conjugated anti-cKit (105826), Bv605-conjugated anti-Sca1 (108133), and FITC-conjugated anti-CD41 (133903). 7-AAD (Invitrogen) was used to exclude dead cells. All antibodies were purchased from BioLegend unless otherwise indicated. Cells were sorted on fluorescence-activated cell sorting (FACS) Aria (BD Bioscience) directly into StemSpan SFEM medium (StemCell Technologies). The sorted cells were incubated for 1 h at 37°C and 5% CO_2_ prior to stimulation with TPO as described above.

### HDAC inhibitor treatment

HPC-7 cells were starved as described above. Cells were pretreated with HDAC inhibitors, 1 µM of Trichostatin A, or 5 nM of Romidepsin (Selleckchem) during the final 30 min of serum starvation and stimulated with TPO at 100 ng/mL for 30 min.

### STAT5 expression

Retrovirus was produced using Phoenix cells (ATCC). HPC-7 cells were infected with retrovirus expressing STAT5B or mutant (Y699F) STAT5B in the presence 8 µg/mL polybrene (Millipore). After 24 h, GFP-positive cells were FACS sorted and cultured in IMDM media (Invitrogen) supplemented with 10% fetal bovine serum and 10% SCF conditioned media.

### RNA preparation and RT-qPCR

Total RNA was isolated using Direct-zol (Zymo Research) and reverse transcribed into cDNA using SuperScript IV First Strand Synthesis System (Invitrogen) following the manufacturer's instructions. Quantitative RT-PCR was carried out using SYBR Green Brilliant II low Rox and a Stratagene Mx3000P machine (Agilent Technologies). For primer sequences, see Supplemental Table S2.

### Subcellular RNA isolation

Subcellular fractionation and RNA preparation were performed essentially as described (Bhatt et al. 2012) with modifications detailed in Supplemental Methods.

### Chromatin immunoprecipitation

Chromatin immunoprecipitation (ChIP) assays on serum-starved and TPO-stimulated HPC-7 cells were performed as previously described (Supplemental Methods; Wilson et al. 2010).

### Hi-C

Hi-C libraries were generated essentially as described (Schoenfelder et al. 2015) with modifications detailed in Supplemental Methods.

### Promoter Capture Hi-C

Promoter Capture Hi-C libraries were generated essentially as described (Schoenfelder et al. 2015) with modifications detailed in Supplemental Methods.

### Computational analyses

Subcellular RNA-seq, ChIP-seq, Hi-C, and promoter Capture Hi-C data processing and all computational analyses are detailed in Supplemental Methods.

## Data access

RNA-seq, ChIP-seq, Hi-C, and promoter Capture Hi-C raw and processed data from this study have been submitted to the NCBI Gene Expression Omnibus (GEO; http://www.ncbi.nlm.nih.gov/geo/) under accession number GSE100835.

## Supplementary Material

Supplemental Material
