# Supplementary material for: Thrombopoietin signaling to chromatin elicits rapid and pervasive epigenome remodeling within poised chromatin architectures
Source: Genome Res. 2018 Mar;28(3):295–309. doi: 10.1101/gr.227272.117 (PMC5848609; doi:10.1101/gr.227272.117)
Supplement: Supplemental Material [file supp_28_3_295__index.html]

Thrombopoietin signaling to chromatin elicits rapid and pervasive epigenome remodeling within poised chromatin architectures — Supplemental Material 

# Thrombopoietin signaling to chromatin elicits rapid and pervasive epigenome remodeling within poised chromatin architectures

## Supplemental Material

- Supplemental\_Fig\_S1\_S7.pdf
- Supplemental\_Methods.pdf
- Supplemental\_Table\_S1.pdf
- Supplemental\_Table\_S2.pdf
