# Supplementary material for: Thrombopoietin signaling to chromatin elicits rapid and pervasive epigenome remodeling within poised chromatin architectures
Source: Genome Res. 2018 Mar;28(3):295–309. doi: 10.1101/gr.227272.117 (PMC5848609; doi:10.1101/gr.227272.117)
Supplement: Supplemental Material [file supp_gr.227272.117_Supplemental_Fig_S1_S7.pdf]

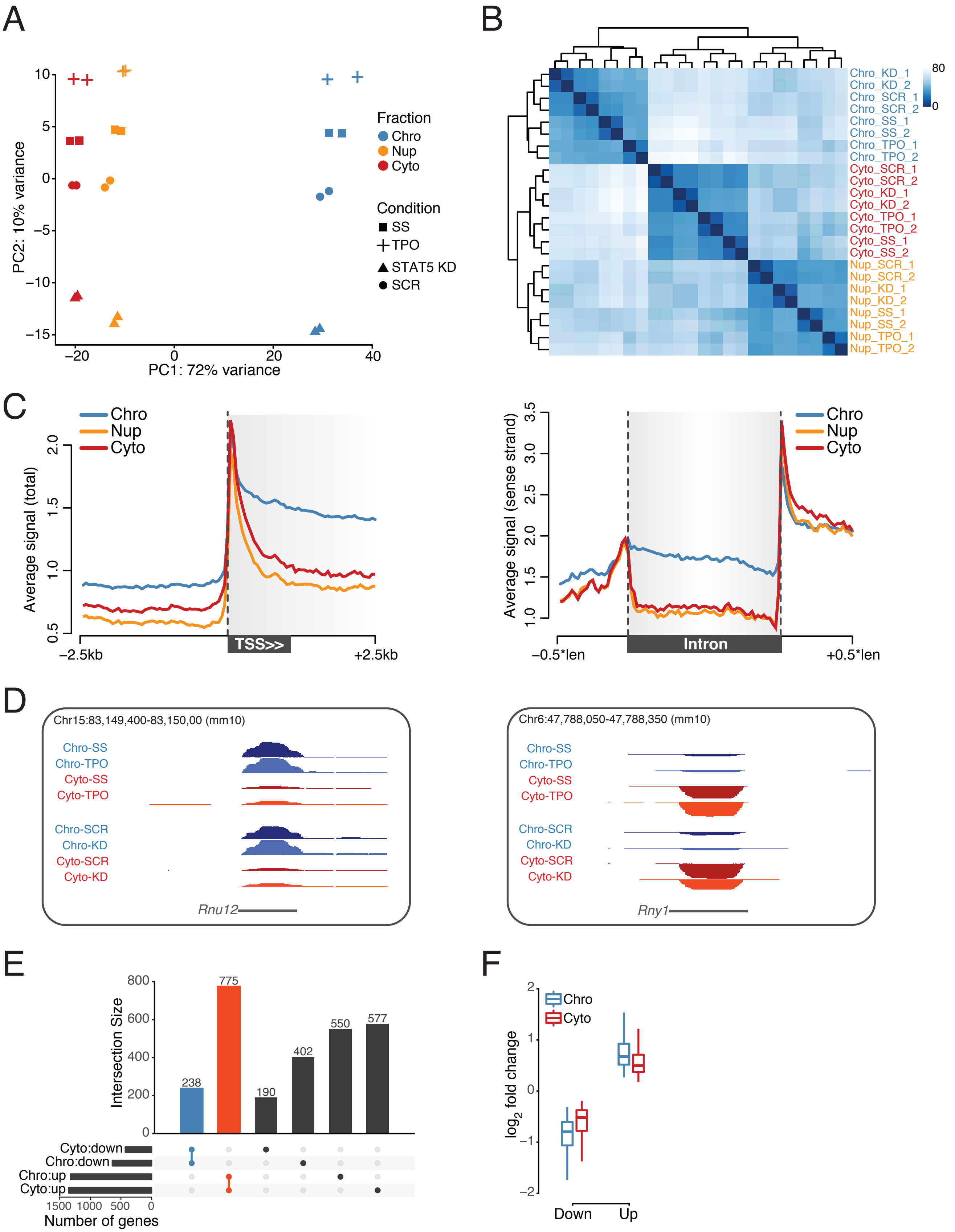
A
B
C
D
E
F
PC1: 72% variance
PC2: 10% variance
Fraction
Chro
Nup
Cyto
Condition
SS
TPO
STAT5 KD
SCR
Chro_KD_1
Chro_KD_2
Chro_SCR_1
Chro_SCR_2
Chro_SS_1
Chro_SS_2
Chro_TPO_1
Chro_TPO_2
Cyto_SCR_1
Cyto_SCR_2
Cyto_KD_1
Cyto_KD_2
Cyto_TPO_1
Cyto_TPO_2
Cyto_SS_1
Cyto_SS_2
Nup_SCR_1
Nup_SCR_2
Nup_KD_1
Nup_KD_2
Nup_SS_1
Nup_SS_2
Nup_TPO_1
Nup_TPO_2
Average signal (total)
TSS>>
–2.5kb
+2.5kb
Average signal (sense strand)
Intron
–0.5*len
+0.5*len
Chr15:83,149,400-83,150,00 (mm10)
Chro-SS
Chro-TPO
Cyto-SS
Cyto-TPO
Chro-SCR
Chro-KD
Cyto-SCR
Cyto-KD
Rnu12
Chr6:47,788,050-47,788,350 (mm10)
Rny1
Intersection Size
238
775
190
402
550
577
Cyto:down
Chro:down
Chro:up
Cyto:up
Number of genes
log2 fold change
Down
Up

Supplemental Figure S1. **(A)** Principal component analysis of gene expression profiles (regularized $\log_2$ expression values) measured by subcellular RNA-seq. Only genes with non-zero counts across all samples were considered. The percentage of variance explained by the first two principal components (PC) is indicated. SS, serum-starved (basal condition); KD, knockdown; SCR, scrambled shRNA control. **(B)** Hierarchical clustering of subcellular RNA-seq samples based on Euclidean distance of gene expression profiles. Only genes with non-zero counts across all samples were considered. **(C)** Average normalized chromatin-associated (Chro), nucleoplasmic (Nup) and cytoplasmic (Cyto) RNA-seq signal at isolated TSSs and introns exhibiting no feature overlap within the indicated genomic windows. **(D)** Representative tracks of chromatin-associated (Chro) and cytoplasmic (Cyto) RNA-seq signals at small nuclear and small cytoplasmic RNA loci (*Rnu12*, U12 small nuclear RNA; *Rny1*, Y1 small cytoplasmic RNA). **(E)** Summary of transcriptional changes induced by 30 minutes TPO stimulation of HPC7 cells. The intersection between differentially transcribed (chromatin-associated RNA-seq) and differentially expressed (cytoplasmic RNA-seq) genes is shown. **(F)** $\log_2$ fold change distribution of differentially transcribed (chromatin-associated RNA-seq) and differentially expressed (cytoplasmic RNA-seq) genes.

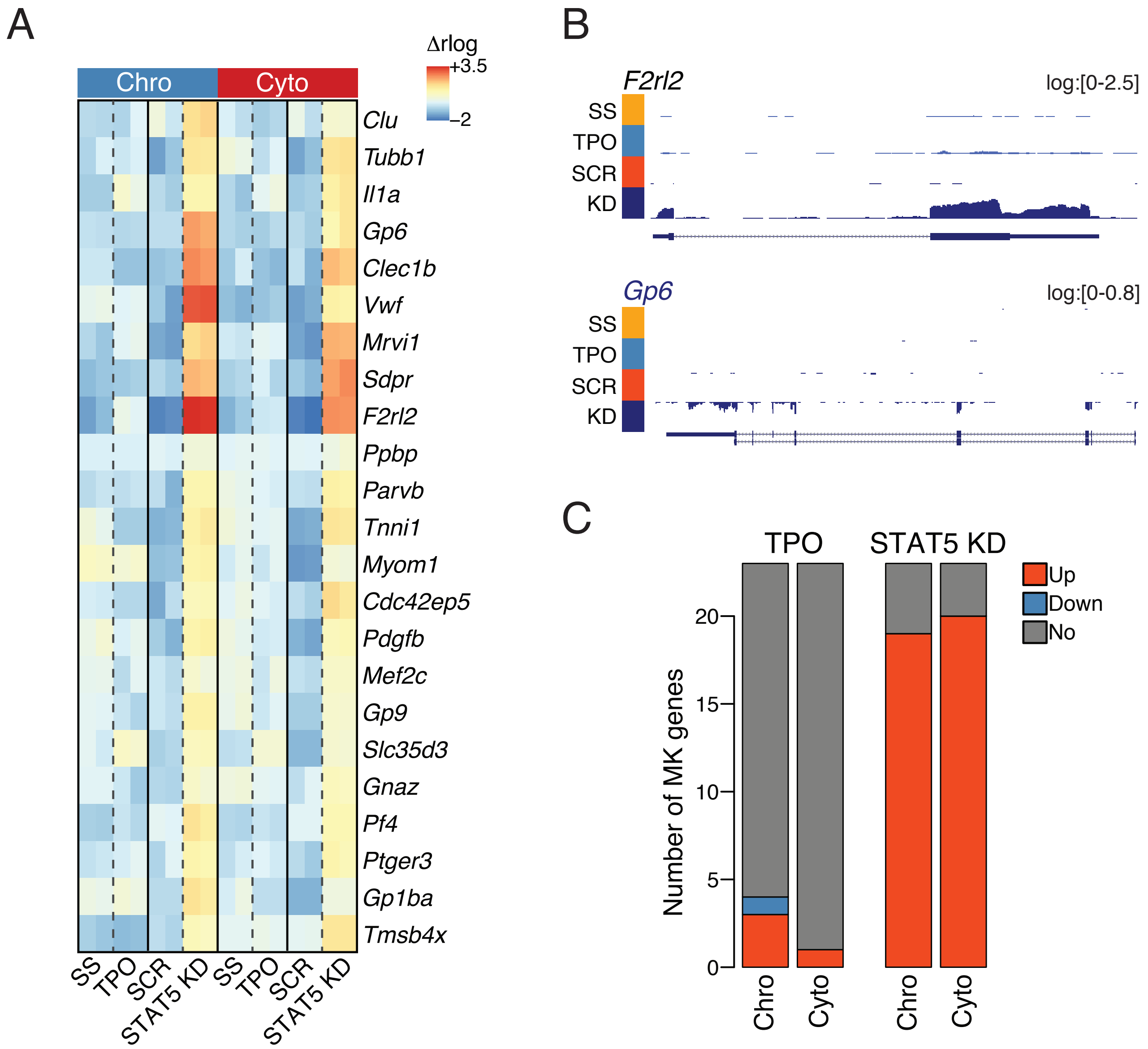

Supplemental Figure S2. **(A)** Chromatin-associated (Chro) and cytoplasmic (Cyto) RNA expression heatmap for megakaryocytic genes upregulated upon shRNA-mediated STAT5 knockdown (KD) in HPC7 cells (Park et al. 2015), for the indicated conditions. Regularized $\log_2$ (rlog) expression values are row-mean subtracted. SCR, scrambled shRNA control. **(B)** Representative tracks of chromatin-associated RNA expression at megakaryocytic genes. RNA-seq coverage was log transformed with a pseudocount of 1. **(C)** Summary of megakaryocytic gene expression changes induced by 30 minutes TPO stimulation, compared to STAT5 knockdown (KD), from chromatin-associated (Chro) and cytoplasmic (Cyto) RNA fractions.

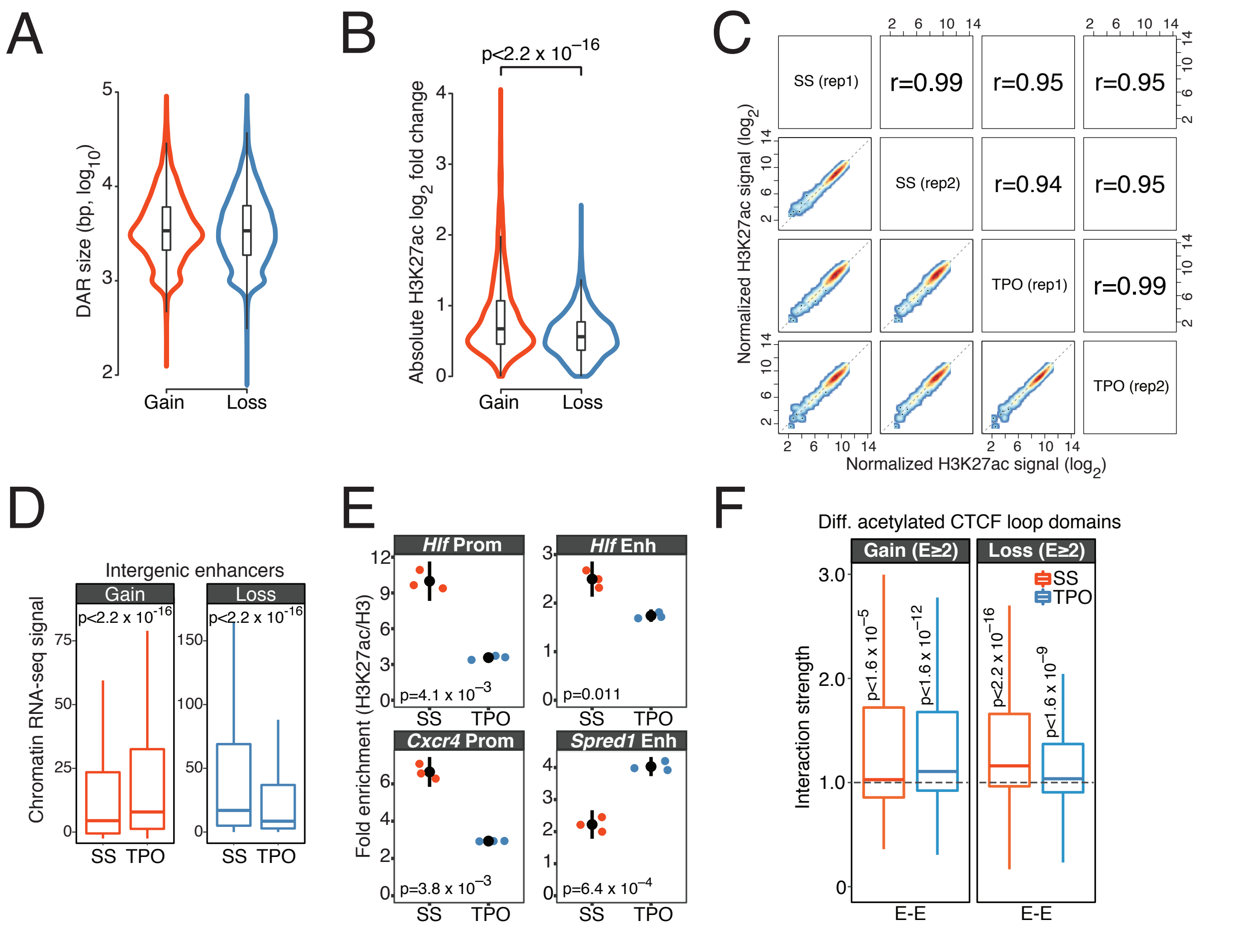

Supplemental Figure S3. **(A)** Size distribution of activated (Gain) and repressed (Loss) DARs. **(B)** H3K27ac $\log_2$-fold change distribution for activated (Gain) and repressed (Loss) DARs. **(C)** Normalized H3K27ac signals within 5 kb windows centered on the transcription start site of the 1,000 most expressed genes in basal condition. Only genes that are not significantly differentially transcribed upon TPO were considered. Spearman's rank correlation coefficients (r) are shown. **(D)** Normalized chromatin-associated RNA expression at differentially acetylated intergenic enhancers. p-values are from a Wilcoxon rank sum test. **(E)** H3K27ac levels normalized to total H3 occupancy at the indicated cis-regulatory elements (see Supplemental Table S2), measured by ChIP-qPCR. Error bars are mean±SD (n=3). p-values are from a two-sided Welch's *t*-test. **(F)** Structured interaction matrix analysis (SIMA) of enhancer-enhancer interactions between CTCF loops located within 20 Mb blocks. The interaction strength reflects the enrichment of Hi-C interactions relative to randomly sampled genomic regions. p-values are from a Wilcoxon signed rank test.

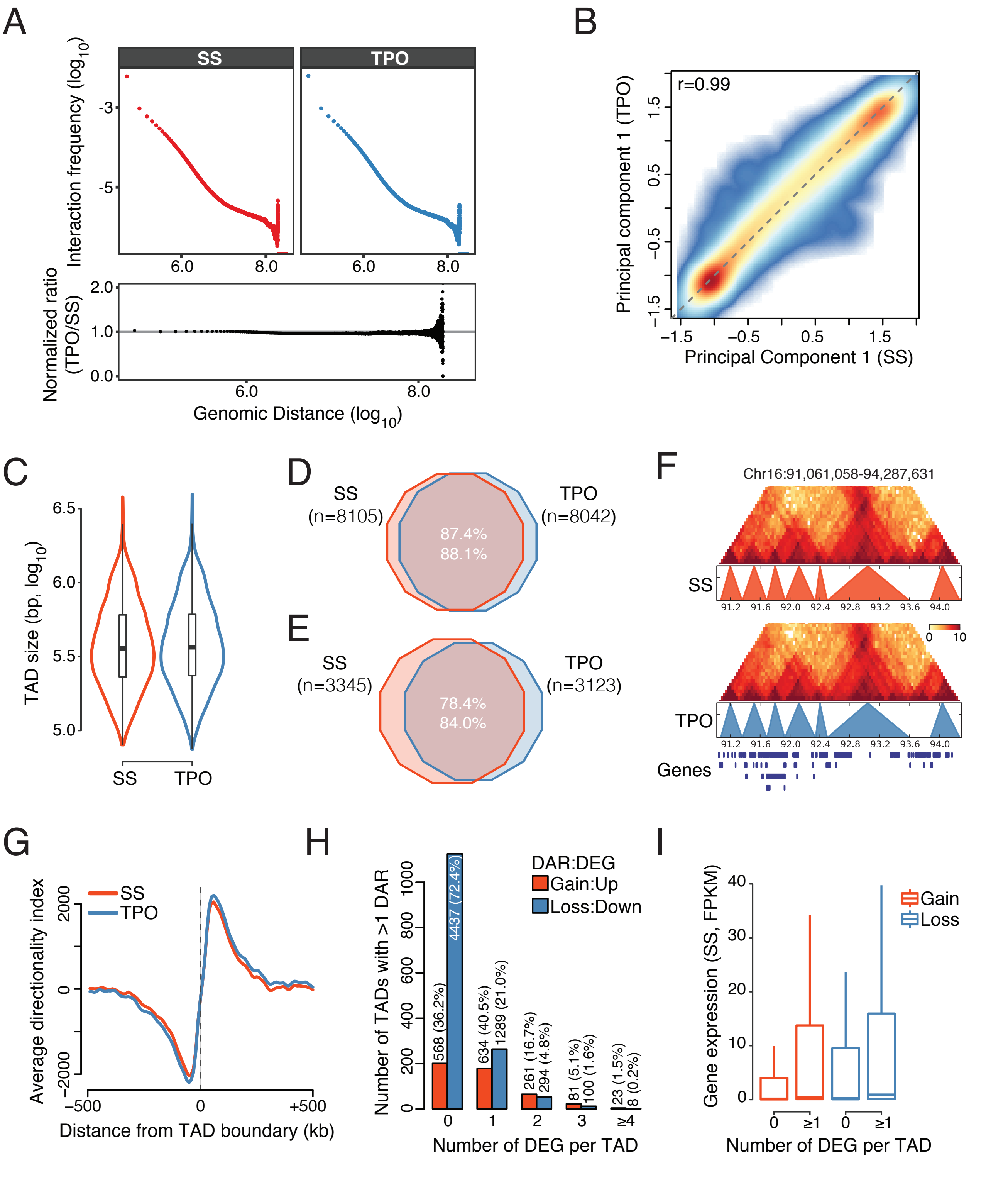

Supplemental Figure S4. **(A)** Intrachromosomal interaction frequency as a function of genomic distance (top) and corresponding normalized interaction frequency ratio between conditions (bottom). **(B)** Genome-wide comparison of PC1 values defining A/B compartments identified in basal condition (SS) and after 30 minutes TPO stimulation. Principal component analysis was performed

on normalized Hi-C contact matrices binned at 40 kb resolution. The Spearman's rank correlation coefficient (r) is shown. **(C)** Size distribution of all detected TADs. **(D)** TAD boundary overlap (maximum tolerance of 10 kb) for all detected TADs. **(E)** Overlap between CTCF loops detected in basal condition (SS) and after 30 minutes TPO stimulation (maximum tolerance of 10 kb). **(F)** A representative 3.2 Mb genomic region (containing *Runx1*) depicting ICE (Iterative Correction and Eigenvector decomposition) normalized Hi-C signals at 40 kb resolution and TAD calls in basal condition (SS) and after 30 minutes TPO stimulation. **(G)** Average directionality index within $\pm$500 kb of a TAD boundary. **(H)** Number of TADs with at least two DARs, partitioned by the number of differentially transcribed genes (DEG) therein. The total number and percentage of DARs within this set of TADs is indicated. **(I)** Distribution of basal gene expression levels (cytoplasmic RNA-seq) of genes located within the TADs in (H). FPKM, fragments per kb of exon per million reads mapped.

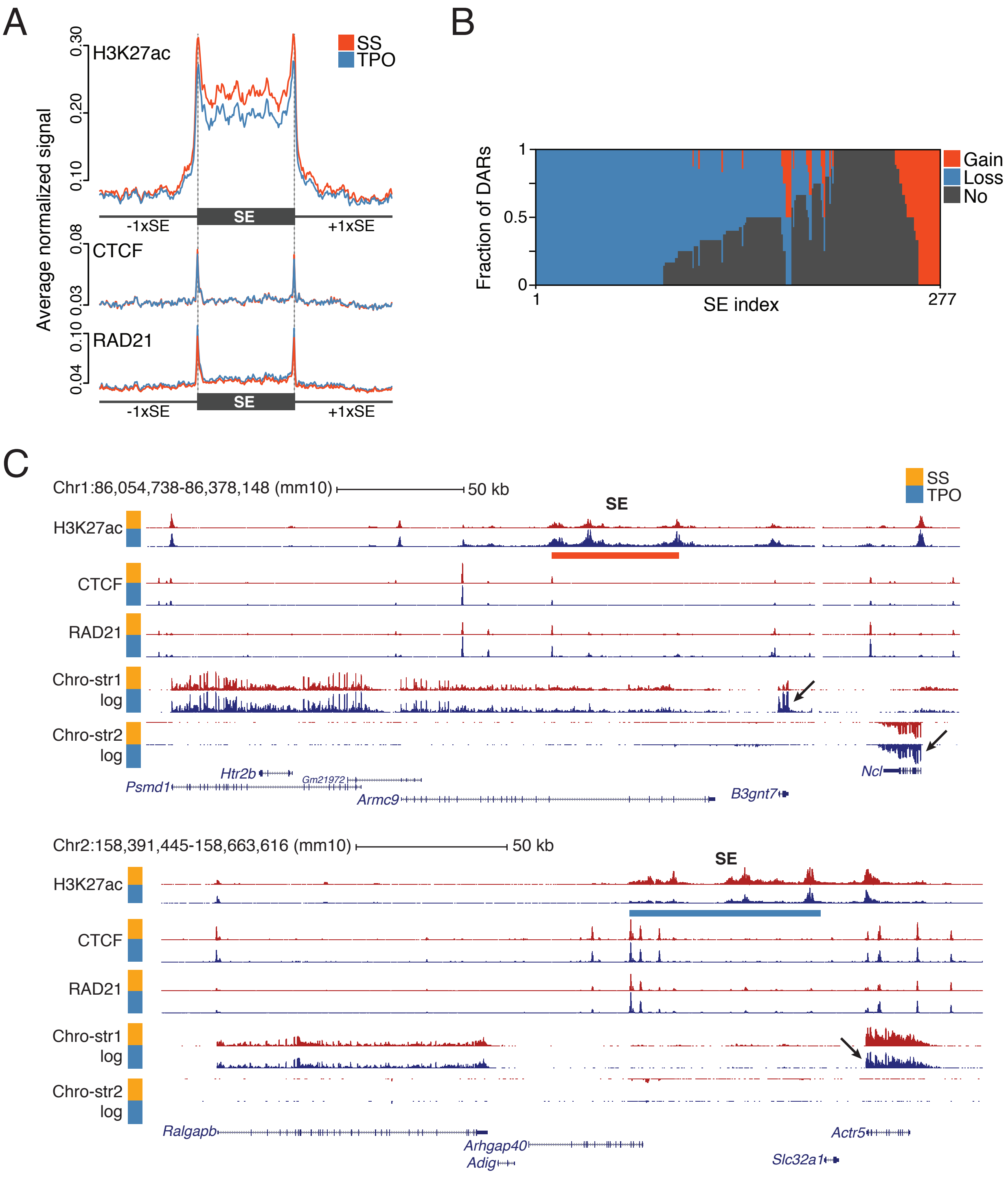

Supplemental Figure S5. **(A)** Average normalized H3K27ac, CTCF and RAD21 ChIP-seq signals at SEs, including flanking regions of size equal to the SE length. Each genomic window was divided into 300 bins. **(B)** Distribution of DARs within SE. Each SE corresponds to a vertical line. **(C)** Representative tracks of differentially transcribed SE-proximal genes. RNA-seq coverage was log transformed with a pseudocount of 1.

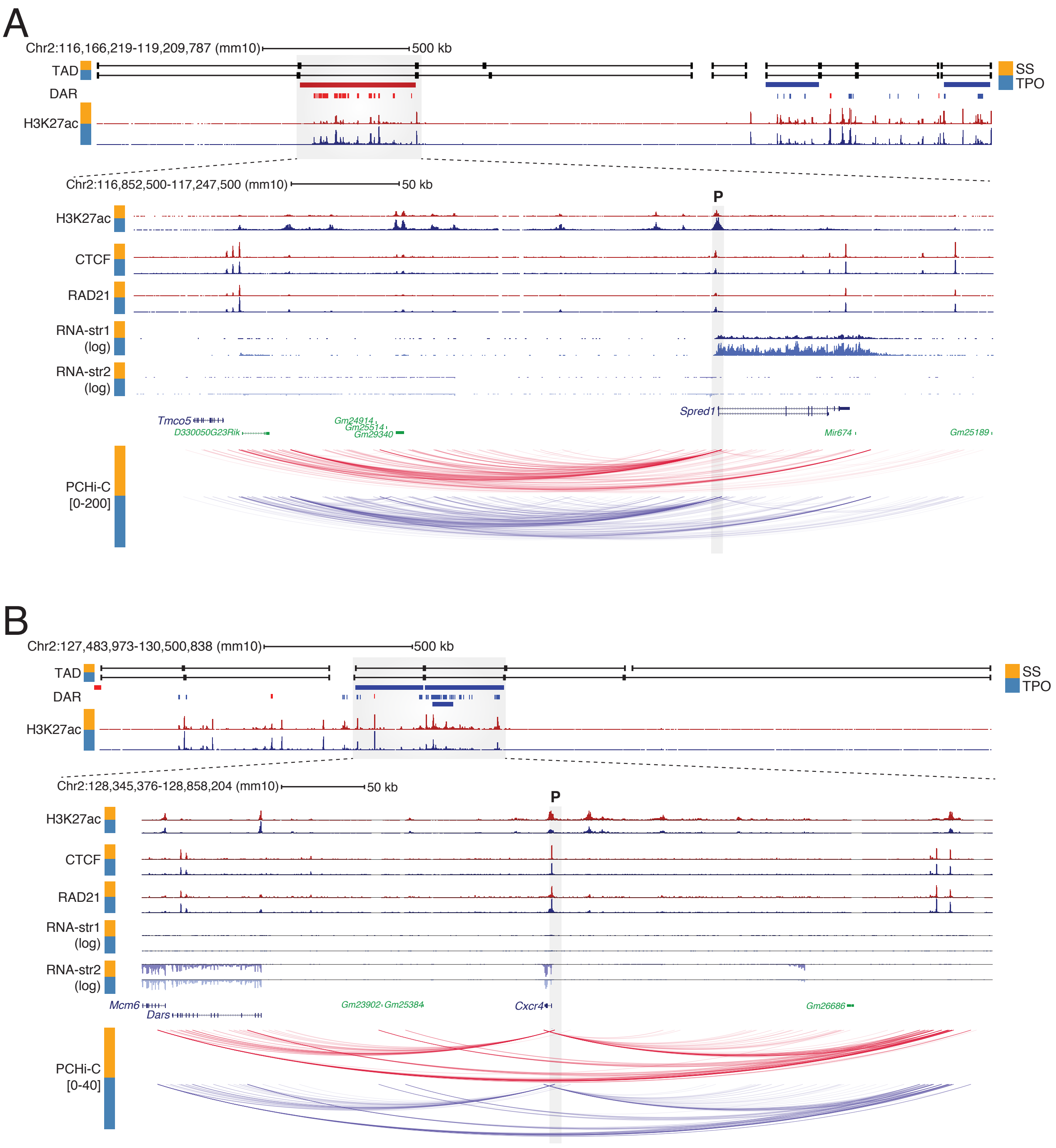

Supplemental Figure S6. **(A)** Epigenomic configuration of the *Spred1* locus. Statistically significant promoter Capture Hi-C interactions within the *Spred1* TAD are shown. The gray shaded rectangle denotes the position of the baited *Spred1* promoter (P). str, strand. **(B)** Same as (A), for the *Cxcr4* locus.

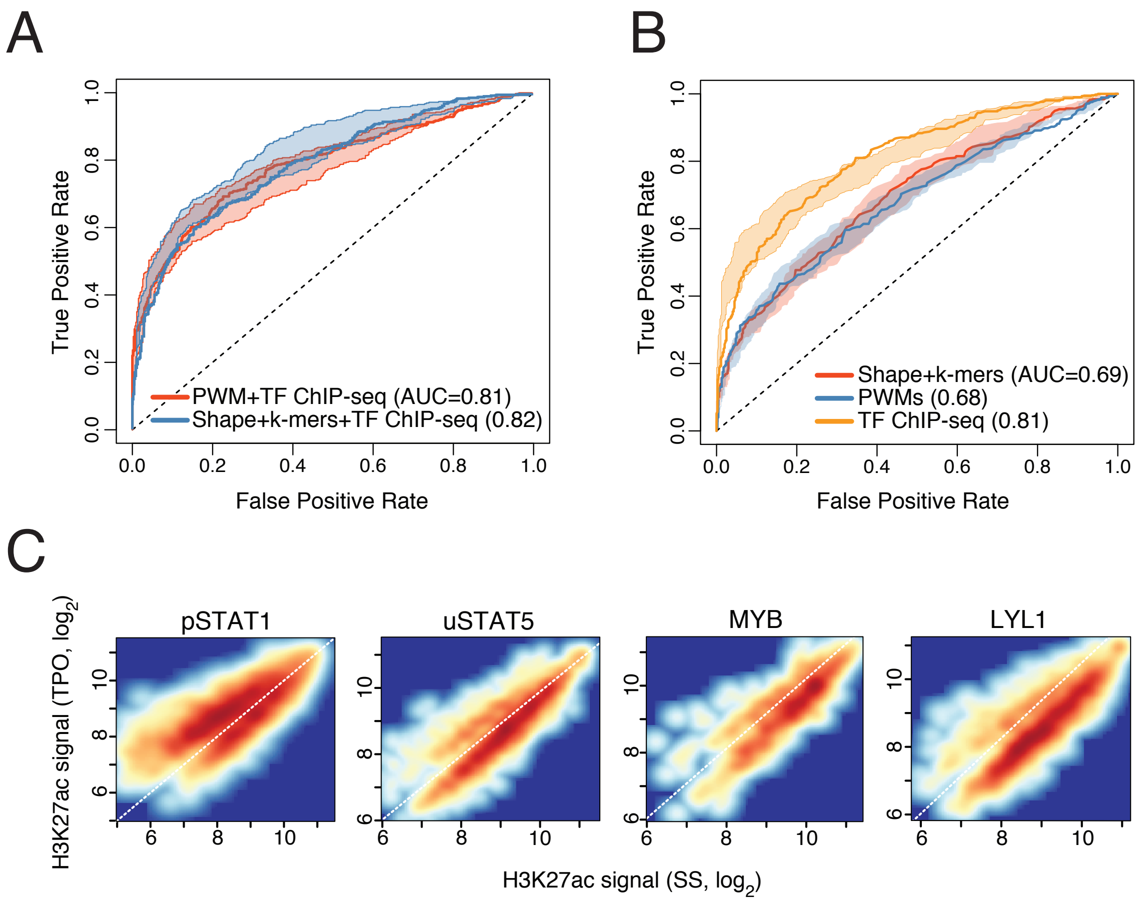

Supplemental Figure S7. **(A)** Test set receiver operating characteristic (ROC) curve and area under the ROC curve (AUC) values for lasso models trained on the indicated sets of features. The shaded area is delimited by the ROC curves for models with highest and lowest AUC values, whereas the ROC curve for the model closest to mean AUC is shown. **(B)** Same as (A), for random forest classifiers. **(C)** Average normalized H3K27ac signals within ±4 kb of the summit of differentially acetylated DHSs bound by the indicated TFs. pSTAT1, tyrosine-phosphorylated STAT1; uSTAT5, tyrosine unphosphorylated STAT5.
