# Supplementary material for: Thrombopoietin signaling to chromatin elicits rapid and pervasive epigenome remodeling within poised chromatin architectures
Source: Genome Res. 2018 Mar;28(3):295–309. doi: 10.1101/gr.227272.117 (PMC5848609; doi:10.1101/gr.227272.117)
Supplement: Supplemental Material [file supp_gr.227272.117_Supplemental_Methods_.pdf]

Supplemental Methods

Subcellular RNA isolation

Subcellular fractionation and RNA preparation were performed essentially as described (Bhatt et al. 2012) with minor modifications. 1×10^6 cells were serum starved and stimulated with TPO as above. Cells were collected by centrifugation and washed once with PBS. The cell pellet was resuspended in ice-cold NP-40 lysis buffer (10 mM Tris-HCl pH 7.5, 0.15% NP40, 150 mM NaCl). The lysate was then layered on 2.5 volumes of a sucrose buffer and centrifuged for 10 min at 13,000 rpm at 4ºC. The supernatant (cytoplasmic fraction) was collected and added 3.5X volumes of RLT Buffer (Qiagen). The nuclei pellet was gently rinsed with ice-cold 1X PBS and resuspended in 200µl ice-cold glycerol buffer (20 mM Tris-HCl pH 7.9, 75 mM NaCl, 0.5 mM EDTA, 0.85 mM DTT, 0.125 mM PMSF, 50% glycerol) by gently flicking the tube. An equal volume of ice-cold nuclei lysis buffer (10 mM HEPES pH 7.6, 1 mM DTT, 7.5 mM $MgCl_2$, 0.2 mM EDTA, 0.3 M NaCl, 1 M UREA, 1% NP-40) was added and gently vortexed twice for 2 sec, incubated for 2 min on ice, and then centrifuged at 13,000 rpm for 2 min at 4°C. The supernatant (nucleoplasmic fraction) was collected and added 3.5X volumes of RLT (Qiagen). The chromatin pellet was gently rinsed with cold 1X PBS and then dissolved in 500 µl TRIzol (Invitrogen). RNA purification from RLT-dissolved samples was performed using RNeasy columns (Qiagen). Chromatin-associated RNA was purified using Direct-zol (Zymo Research).

Chromatin immunoprecipitation

Chromatin immunoprecipitation (ChIP) assays on serum-starved and TPO stimulated HPC-7 cells were performed as previously described (Wilson et al. 2010). Briefly, 1×10^8 cells were cross-linked in 1% formaldehyde for 10 min at room temperature. Cells were lysed in 10mM Tris pH 8.0, 10mM NaCl and 0.2% NP40 containing inhibitors (1µg/mL leupeptin, 10mM NaBu and 50µg/mL PMSF) for 10 min on ice and were collected by centrifugation at 2500 rpm for 5 min at 4ºC. The nuclei pellet was frozen until further use.

Frozen nuclei were resuspended in 50mM Tris pH 8.0, 10mM EDTA, 1% SDS supplemented with inhibitors (1µg/mL leupeptin, 10mM NaBu and 50µg/mL PMSF)

and sonicated in an equal volume of IP dilution buffer (20mM Tris pH 8.0, 2mM EDTA, 150mM NaCl, 1% Triton X-100, 0.01% SDS and protease inhibitors) in ice-water (Bioruptor, Diagenode) for 5 cycles (30s on, 30s off). Chromatin was pre-cleared with non-specific rabbit IgG (2 µg/µl, Sigma) for 1 hour and 100µL of protein G Dynabeads (Invitrogen) for 2 hours. The beads/IgG were removed by magnetic separation. Chromatin was immunoprecipitated at 4ºC overnight using antibodies against H3K27ac (Abcam, 4729), histone H3 (Abcam, 1791), RAD21 (Abcam, 992), CTCF (Millipore, 07-729), STAT1 (Cell Signaling, 9172) or a control rabbit IgG (Invitrogen, 9172) and 100µl of protein G Dynabeads (Invitrogen) were added for additional 2 hours. Immunocomplexes were washed and eluted twice from the beads with 150µL elution buffer (100mM NaHCO3, 1% SDS). Cross-linking was reversed overnight with 0.3M NaCl and 2µL of RNase (10mg/mL) at 65ºC, and samples were further treated with Proteinase K (20mg/mL) for 2 hours at 42ºC. The ChIP DNA was purified using a PCR purification kit (Qiagen).

Hi-C

Hi-C libraries were generated essentially as described (Schoenfelder et al. 2015) with modifications detailed below. 3.5×10^7 HPC-7 cells were fixed in 2% formaldehyde (Agar Scientific) for 10 min, after which the reaction was quenched with ice-cold glycine (Sigma; 0.125M final concentration). Cells were collected by centrifugation (400 x g for 10 min at 4ºC), and washed once with 50 mL PBS pH 7.4 (Gibco). After another centrifugation step (400 x g for 10 min at 4ºC), the supernatant was completely removed and the cell pellets were immediately frozen in liquid nitrogen and stored at -80˚C.

After thawing, the cell pellets were incubated in 50 mL ice-cold lysis buffer (10 mM Tris-HCl pH 8, 10 mM NaCl, 0.2% Igepal CA-630, protease inhibitor cocktail (Roche)) for 30 min on ice. After centrifugation to pellet the cell nuclei (650 x g for 5 min at 4ºC), nuclei were washed once with 1.25 x NEBuffer 2 (NEB). The nuclei were then resuspended in 1.25 x NEBuffer 2, SDS (10% stock; Promega) was added (0.3% final concentration) and the nuclei were incubated at 37ºC for one hour with agitation (950 rpm). Triton X-100 (Sigma) was added to a final concentration of 1.7 % and the nuclei were incubated at 37ºC for one hour with agitation (950 rpm). Restriction digest was performed overnight at 37ºC with agitation (950 rpm) with *HindIII* (NEB; 1500 units per

7 million cells). Using biotin-14-dATP (Life Technologies), dCTP, dGTP and dTTP (Life Technologies; all at a final concentration of 30 µM), the *HindIII* restriction sites were then filled in with Klenow (NEB) for 75 minutes at 37ºC, followed by ligation for 4 hours at 16ºC (50 units T4 DNA ligase (Life Technologies) per 7 million cells starting material) in a total volume of 5.5 mL ligation buffer (50 mM Tris-HCl, 10 mM $MgCl_2$, 1 mM ATP, 10 mM DTT, 100 µg/mL BSA, 0.9 % Triton X-100) per 7 million cells starting material. After ligation, crosslinking was reversed by incubation with Proteinase K (Roche; 65 µl of 10mg/mL per 7 million cells starting material) at 65˚C overnight. An additional Proteinase K incubation (65 µl of 10mg/mL per 7 million cells starting material) at 65ºC for two hours was followed by RNase A (Roche; 15 µl of 10mg/mL per 7 million cells starting material) treatment and two sequential phenol/chloroform (Sigma) extractions. After DNA precipitation (sodium acetate 3M pH 5.2 (1/10 volume) and ethanol (2.5 x volumes)) overnight at -20ºC, the DNA was spun down (centrifugation 3200 x g for 30 min at 4ºC). The pellets were resuspended in 400 µl TLE (10 mM Tris-HCl pH 8.0; 0.1 mM EDTA), and transferred to 1.5 mL eppendorf tubes. After another phenol/chloroform (Sigma) extraction and DNA precipitation overnight at -20ºC, the pellets were washed thrice with 70% ethanol, and the DNA concentration was determined using Quant-iT Pico Green (Life Technologies). For quality control, candidate 3C interactions were assayed (Supplemental Table S2) by PCR, and the efficiency of biotin incorporation was assayed by amplifying a 3C ligation product (Supplemental Table S2), followed by digest with *HindIII* or *NheI*.

To remove biotin from non-ligated fragment ends, 40 µg of Hi-C library DNA were incubated with T4 DNA polymerase (NEB) for 4 hours at 20ºC, followed by phenol/chloroform purification and DNA precipitation overnight at -20ºC. After one wash with 70% ethanol, sonication was carried out to generate DNA fragments with a size peak around 400 bp (Covaris E220 settings: duty factor: 10%; peak incident power: 140W; cycles per burst: 200; time: 55 seconds). After end repair (T4 DNA polymerase, T4 DNA polynucleotide kinase, Klenow (all NEB) in the presence of dNTPs in ligation buffer (NEB)) for 30 min at room temperature, the DNA was purified (Qiagen PCR purification kit). dATP was added with Klenow exo- (NEB) for 30 min at 37ºC, after which the enzyme was heat-inactivated (20 min at 65ºC). A double size selection using AMPure XP beads (Beckman Coulter) was performed: first, the ratio of AMPure XP beads solution volume to DNA sample volume was adjusted to 0.6:1. After incubation for 15 min at room temperature, the sample was transferred to a

magnetic separator (DynaMag-2 magnet; Life Technologies), and the supernatant was transferred to a new eppendorf tube, while the beads were discarded. The ratio of AMPure XP beads solution volume to DNA sample volume was then adjusted to 0.9:1 final. After incubation for 15 min at room temperature, the sample was transferred to a magnet (DynaMag-2 magnet; Life Technologies). Following two washes with 70% ethanol, the DNA was eluted in 100 µl of TLE (10 mM Tris-HCl pH 8.0; 0.1 mM EDTA). Biotinylated ligation products were isolated using MyOne Streptavidin C1 Dynabeads (Life Technologies) on a DynaMag-2 magnet (Life Technologies) in binding buffer (5 mM Tris pH8, 0.5 mM EDTA, 1 M NaCl) for 30 min at room temperature. After two washes in binding buffer and one wash in ligation buffer (NEB), PE adapters (Illumina) were ligated onto Hi-C ligation products bound to streptavidin beads for 2 hours at room temperature (T4 DNA ligase NEB, in ligation buffer, slowly rotating). After washing twice with wash buffer (5 mM Tris, 0.5 mM EDTA, 1 M NaCl, 0.05% Tween-20) and then once with binding buffer, the DNA-bound beads were resuspended in a final volume of 90 µl NEBuffer 2. Bead-bound Hi-C DNA was amplified with 7 PCR amplification cycles using PE PCR 1.0 and PE PCR 2.0 primers (Illumina). After PCR amplification, the Hi-C libraries were purified with AMPure XP beads (Beckman Coulter). The concentration of the Hi-C libraries was determined by Bioanalyzer profiles (Agilent Technologies), and the Hi-C libraries were paired-end sequenced (HiSeq 4000, Illumina).

Promoter Capture Hi-C

Promoter Capture Hi-C libraries were generated essentially as described (Schoenfelder et al. 2015) with modifications detailed below. 500 ng of Hi-C library DNA was resuspended in 3.6 µl H_2O, and hybridization blockers (Agilent Technologies; hybridization blockers 1 and 2, and custom hybridization blocker) were added to the Hi-C DNA. Hybridization buffers and the custom-made RNA capture bait system (Agilent Technologies; designed as previously described (Schoenfelder et al. 2015): 39,021 individual biotinylated RNAs targeting the ends of 22,225 promoter-containing mouse *HindIII* restriction fragments) were prepared according to the manufacturer's instructions (SureSelect Target Enrichment, Agilent Technologies). The Hi-C library DNA was denatured for 5 min at 95ºC, and then incubated with hybridization buffer and the RNA capture bait system at 65ºC for 24 hours (all incubation steps in a MJ Research PTC-200 PCR machine). After hybridization, 60 µl

of MyOne Streptavidin T1 Dynabeads (Life Technologies) were washed thrice with 200 µl binding buffer (SureSelect Target Enrichment, Agilent Technologies), before incubation with the Hi-C DNA/RNA capture bait mixture with 200 µl binding buffer for 30 min at room temperature, slowly rotating. Hi-C DNA bound to capture RNA was isolated using a DynaMag-2 magnet (Life Technologies). Washes (15 min in 500 µl wash buffer I at room temperature, followed by three 10 min incubations in 500 µl wash buffer II at 65ºC) were performed according to the SureSelect Target enrichment protocol (Agilent Technologies). After the final wash, the beads were resuspended in 300 µl NEBuffer 2, isolated on a DynaMag-2 magnet, and then resuspended in a final volume of 30 µl NEBuffer 2. After a post-capture PCR (four amplification cycles using Illumina PE PCR 1.0 and PE PCR 2.0 primers; 13 to 15 individual PCR reactions), the Promoter CHi-C libraries were purified with AMPure XP beads (Beckman Coulter). The concentration of the Promoter CHi-C libraries was determined by Bioanalyzer profiles (Agilent Technologies), and the Promoter CHi-C libraries were paired-end sequenced (HiSeq 4000, Illumina).

RNA-seq data analysis

Subcellular RNA-seq reads were quality and adapter trimmed using Trimmomatic v0.33 (Bolger et al. 2014) in palindrome mode (Illumina TruSeq universal adapter sequence). Reads were aligned to the mouse reference genome (GRCm38 primary assembly, release 82) using STAR v2.4.2a (Dobin et al. 2013). Gene-level count tables were generated while mapping based on Gencode vM7 annotations. Normalized (reads per million, RPM) strand-specific bedGraph tracks were generated while mapping and converted to bigWig format using BEDTools v2.17.0 (Quinlan and Hall 2010). For visualization, the RNA-seq coverage was log transformed with a pseudocount of 1 where indicated.

Downstream analyses were performed using R v3.3.2 and Bioconductor (Huber et al. 2015). Differentially transcribed and differentially expressed genes were identified using DESeq2 v1.14.1 (Love et al. 2014). False discovery rates were controlled at a 0.1% level by applying an independent hypothesis weighting (IHW) procedure (Ignatiadis et al. 2016) using the mean of normalized counts for each gene as the informative covariate. Conditional independence of p-values under the null hypothesis was verified prior to IHW.

ChIP-seq data analysis

ChIP-seq reads were aligned to the mouse reference genome (GRCm38 primary assembly, release 82) using Bowtie2 v2.2.3 (Langmead and Salzberg 2012), allowing no mismatch within a seed of 22 nt. SAM files were converted to BAM format, sorted, de-duplicated and indexed with Sambamba v0.6.5 (Tarasov et al. 2015). Reads mapping to unplaced scaffolds or to the mitochondrial genome were discarded. Downstream analyses were performed using Homer v4.9 (Heinz et al. 2010). Uniquely aligned reads were used to generate Homer tag directories, and peak calling was performed using the findPeaks function. For H3K27ac ChIP-seq profiles, peaks were called against matched IgG control profiles in histone mode with a 1 kb window size and a 2.5 kb distance cutoff. High-confidence peak regions were defined as the peak intersection between biological replicates (computed with BEDTools v2.17.0, minimum overlap of 1 bp). Normalized (RPM) bigWig tracks were generated using the makeMultiWigHub.pl script.

Classification of cis-regulatory elements

DNaseI-seq data (Wilson et al. 2016) were aligned to the mm10 mouse reference genome with Bowtie2 v2.2.3, allowing no mismatch within a 22 nt seed. DNaseI hypersensitive sites (DHS) were called from a pool of four biological replicates using MACS2 v2.1.0 (Zhang et al. 2008) and --no-model option, at 5% FDR. DHS separated by less than 150 bp (approximately one nucleosome) were merged, and H3K4me1 and H3K4me3 signals, defined as offset-normalized enrichments over input, were computed in 1 kb windows centered on the resulting DHS regions. This window size maximized the variance of H3K4me1/H3K4me3 ratios among all tested window sizes in the range (10, 2500) bp. DHS exhibiting an H3K4me1 and H3K4me3 fold enrichment over input < 2 were classified as insulators and were not considered further. Offset-normalized H3K4me1/H3K4me3 ratios X were computed at the remaining DHS regions. To discriminate enhancers from promoters, the distribution of $\log_2(X)$ was fitted by a two-component Gaussian mixture model with unequal variance using mclust v5.1.

Differential acetylation analysis

Differentially acetylated regions (DARs) were called from the union of all high-confidence H3K27ac peaks identified across conditions using csaw v1.8.0 (Lun and

Smyth 2015) with the following parameters: window size, 1000 bp; spacing, 200 bp; fragment length, 200 bp; filter, 10 reads; minimum mapping quality, 10. Differentially acetylated windows were identified by fitting a quasi-likelihood negative binomial model (Lun et al. 2016) and subsequently merged into DARs at 1% FDR. DARs exhibiting heterotypic differentially acetylated windows were discarded. DARs were annotated to genomic compartments and cis-regulatory elements using the ChIPseeker package v1.10.3 (Yu et al. 2015).

Hi-C data analysis

Hi-C reads were pre-processed with HiC-Pro v2.7.7 (Servant et al. 2015). The digest_genome.py utility was used to digest the mouse reference genome (GRCm38 primary assembly, release 82) in silico, generating a BED file containing all theoretical HindIII restriction fragments. HiC-Pro was run with Bowtie2 v2.2.3. The algorithm performs a two-step read alignment to the reference genome whereby reads are first independently aligned using the Bowtie2 end-to-end algorithm, followed by detection of ligation sites on unmapped chimeric reads and re-alignment of their 5' fractions. Singletons, multi-mappers, duplicated reads, and read pairs with mapping quality < 10 were discarded. Aligned reads were assigned to restriction fragments and read pairs corresponding to invalid ligation products (e.g. self-ligations, dangling ends) or mapping outside the insert size range [150, 800], or anchored at unplaced scaffolds or the mitochondrial genome were discarded. After a correlation analysis of Hi-C signals between individual samples, valid pairs from biological replicates were merged. These were used to compute raw and coverage-and-distance corrected ICE (iterative correction and eigenvector decomposition) contact matrices (Imakaev et al. 2012), and to generate Homer tag directories for which signals exceeding 10 times the average read counts in 10 kb bins were removed.

A/B compartments and topological domains were called using Homer as follows. For each chromosome, A/B compartments were identified by computing the first eigenvector of a binned (40kb resolution) interaction profile correlation matrix. Transcription start site coordinates of annotated genes were used to assign active and inactive compartments to positive and negative eigenvector values, respectively. TADs were identified using the Homer Hi-C domain finding algorithm, which computes the ratio of upstream and downstream interaction counts (directionality index, DI) within a given fixed-size window (Dixon et al. 2012). A window size of 1 Mb, a bin size

of 25 kb and a step size of 5 kb were used. Bins exhibiting coverage values smaller than 15% of the mean bin coverage or exceeding it by more than four standard deviations were excluded. DI values were smoothed using a running average over ±25 kb window. Domain boundary coordinates were defined based on smoothed DI profiles, requiring a minimum index score of 0.5. For comparison of TAD coordinates across conditions, a maximum tolerance of 10 kb was allowed at the TAD boundaries. Differentially acetylated TADs were identified using csaw as described above. Differentially acetylated windows were merged into differentially acetylated TADs at 1% FDR, using the genomic coordinates of TADs identified in basal condition and requiring at least 75% homotypic differentially acetylated windows therein.
To identify CTCF loops, statistically significant Hi-C interactions were first called using the Homer analyzeHiC algorithm with parameters -interactions -res 10000 –superRes 20000 -center -nomatrix. Significant interactions were then filtered to retain only intrachromosomal interactions at 5% FDR. Of these, only interactions whose anchors overlapped both a CTCF and a Rad21 peak and exhibited an unambiguous CTCF motif direction were considered. CTCF loops were then defined as CTCF/Rad21-anchored interactions with convergent CTCF motif orientation.

Structured interaction matrix analysis

Enhancer-enhancer interactions within and between differentially acetylated TADs were analyzed using a structured interaction matrix analysis (SIMA) (Lin et al. 2012), which pools Hi-C signals across genomic regions of interest located within a given set of chromatin domains. SIMA was run separately on activated and repressed TADs of size ≥ 200 kb (-minDsize 2e5) to analyze two sets of genomics regions of interest: i) differentially acetylated enhancers (i.e. enhancers located within DARs, resized to 1 kb); ii) a control set of genomic regions obtained by systematically shifting (10 kb downstream) the genomic coordinates of differentially acetylated enhancers. A super-resolution of 10 kb (-superRes 10000; i.e. all reads within a 10 kb window centered on each enhancer were considered) and a resolution of 2.5 kb (-res 2500) were used. Interactions across TADs were analyzed for differentially acetylated TADs separated by ≤ 20 Mb (-max 2e7). The same analysis was performed for CTCF loops.

Promoter Capture Hi-C data analysis

Promoter Capture Hi-C (PCHi-C) reads were pre-processed with HiC-Pro v2.7.7 as described above. Statistically significant PCHi-C interactions were computed with CHiCAGO v1.2.0 (Cairns et al. 2016). To this end, a restriction map file and a bait map file (both in BED format) were used to precompute auxiliary files using the makeDesignFiles.py script from chicagoTools. To generate input files for the CHiCAGO pipeline, HiC-Pro valid pairs in BAM format were mapped to restriction fragments using the HiC-Pro mapped_2hic_fragments.py script, de-duplicated using Sambamba v0.6.5, sorted by natural sort, and converted to CHiCAGO format using the bam2chicago.sh script provided by chicagoTools. CHiCAGO design files were generated using the makeDesignFiles.py script with parameters --minFragLen=150 --maxFragLen=40000 --maxLBrownEst=1500000 --binsize=20000 --removeb2b=True --removeAdjacent=True. An interaction score ≥ 5 was used to call statistically significant PCHi-C interactions. Annotation of PCHi-C interactions to genomic regions was performed using the GenomicInteractions package v1.8.0 (Harmston et al. 2015). To correlate changes in H3K27ac levels with changes in PCHi-C interaction frequency at individual differentially transcribed genes, cis-regulatory units were first defined for each baited promoter by considering the set of all its PIRs across conditions. PIRs were further filtered to retain only regions overlapping at least one high-confidence H3K27ac peak, ensuring stable estimates of H3K27ac ratios between conditions. In addition, cis-regulatory units containing less than five H3K27ac-marked PIRs were discarded, resulting in a set of 907 differentially transcribed loci that were further analyzed as follows. Given a cis-regulatory unit, the normalized H3K27ac fold change and the normalized ratio between PCHi-C signals in TPO and basal conditions (interaction frequency fold change) were computed for each PIR. The correlation between interaction frequency and H3K27ac fold changes was then computed for each cis-regulatory unit using a Spearman's rank correlation coefficient. Statistically significant correlation values were identified by deriving a null distribution for the correlation coefficient. This was estimated using a randomization procedure whereby the observed (H3K27ac fold change, interaction frequency fold change) pairs were randomly permuted 50 times for each cis-regulatory unit and the corresponding Spearman's rank correlation coefficient were recorded. The empirical distribution of all recorded correlation values was used to perform a one-sided statistical test.

Superenhancers

Superenhancers were defined starting from the genomic coordinates of the individual constituent enhancer elements. First, enhancers located within 2.5 kb of annotated transcription start sites or not overlapping an H3K27ac peak were discarded. The remaining enhancers were then stitched together if located closer than 20 kb. This value has been chosen after examining the monotone relation between the number of stitched regions and the distance threshold. Stitched regions were then ranked by total input-normalized H3K27ac signals and classified in superenhancers or regular enhancers as previously described (Whyte et al. 2013).
Differentially acetylated SEs were identified using csaw as described above. Differentially acetylated windows were merged into differentially acetylated SEs at 1% FDR, using the genomic coordinates of SEs identified in basal condition. Differentially acetylated SEs exhibiting heterotypic differentially acetylated windows were discarded.

Motif analysis

De novo motif discovery was performed using Homer (findMotifsGenome.pl script), searching for motifs of length 6-12 nt. For the analysis of differentially acetylated cis-regulatory elements, DHSs located within DARs were considered and 200 nt sequences centered on the DHS summit were extracted. For motif density analysis, PWMs of top TF motif hits and a collection of 363 Homer PWMs for vertebrate TFs were scored within 2kb windows centered on DHS summits at 10 bp resolution using the annotatePeaks.pl script.

Statistical learning

Logistic regression models are a class of probabilistic binary classifiers. Least absolute shrinkage and selection operator (lasso) logistic regression penalizes model complexity through an L1 norm penalty. The lasso generates a sparse model representation through intrinsic feature selection, effectively contrasting overfitting in high-dimensional feature spaces (Tibshirani 1996). Model parameters are usually estimated from a training set of labeled data points using cross-validation, and model performances are evaluated on a test set that has not been previously seen by the model.
Here, lasso logistic regression models were used to predict rapid cis-regulatory responses to TPO signaling. For feature scoring, DNA sequences of differentially

acetylated cis-regulatory elements (defined as promoters and enhancers located within DARs, see above) were extracted from the mm10 reference genome within 200 bp and 500 bp windows centered on the DHS summit. The following features were scored therein:

1. DNA sequence content encoded as *k*-mers ($2 \leq k \leq 4$), computed within 500 bp windows.
2. Average DNA shape feature values within 200 bp windows. These features were computed using the R package DNAshapeR v1.2.0 (Chiu et al. 2016), which implements a high-throughput approach based on all-atom Monte-Carlo predictions. Four DNA shape features were used in this study: helix twist, propeller twist, minor groove width and roll.
3. Transformed FIMO p-values ($-10*\log_{10}(p)$) (Grant et al. 2011) for a curated collection of >1,700 single and composite position weight matrices (PWMs) representing mammalian TF motifs (Diaferia et al. 2016). Only TFs expressed in HPC-7 cells were considered (FPKM ≥ 1 across all cytoplasmic RNA-seq samples), along with composite motifs for expressed TFs. FIMO scores were computed as previously described (Barozzi et al. 2014) using the FIMO implementation provided as part of MEME v4.11.3.
4. ChIP-seq signals for a collection of 29 genome-wide binding profiles for hematopoietic and other sequence-specific TFs in HPC-7 cells (Supplemental Table S1). These profiles were generated as part of this study or previously published (Wilson et al. 2010, 2016). Feature enrichments were computed as previously described (Comoglio et al. 2015) within 1 kb windows centered on the DHS summit.
5. Normalized Hi-C signals defined as the total number of valid di-tags anchored within 1 kb windows centered on the DHS summit. These were used as a proxy for interaction frequencies.

Data points were randomly partitioned into 100 balanced training (80%) and test (20%) sets composed of an equal number of activated and repressed cis-regulatory elements. Lasso logistic regression models were trained on each training set with ten-fold cross validation using the glmnet implementation (Friedman et al. 2010). The value of the regularization parameter that minimized the cross-validated misclassification error was used to predict the class labels of the cis-regulatory

elements in the corresponding test set. Model performances were evaluated by computing the area under the receiver operating characteristic curve (AUC) using the R package ROCR v1.0-7 (Sing et al. 2005) and average AUC values across all 100 models were computed. Feature importance analysis was carried using the bootstrap-lasso algorithm as previously described (Comoglio and Paro 2014; Comoglio et al. 2015). Features with selection probability (stability) ≥ 0.7 were considered.

Random forest classifiers (Breiman 2001) were trained and evaluated on the same balanced training and test sets using the R package randomForest v4.6-12.

Supplemental References

Barozzi I, Simonatto M, Bonifacio S, Yang L, Rohs R, Ghisletti S, Natoli G. 2014. Coregulation of Transcription Factor Binding and Nucleosome Occupancy through DNA Features of Mammalian Enhancers. *Mol Cell* **54**: 844–857.

Bhatt DM, Pandya-Jones A, Tong AJ, Barozzi I, Lissner MM, Natoli G, Black DL, Smale ST. 2012. Transcript dynamics of proinflammatory genes revealed by sequence analysis of subcellular RNA fractions. *Cell* **150**: 279–290.

Bolger AM, Lohse M, Usadel B. 2014. Trimmomatic: A flexible trimmer for Illumina sequence data. *Bioinformatics* **30**: 2114–2120.

Breiman L. 2001. Random forests. *Mach Learn* **45**: 5–32.

Cairns J, Freire-Pritchett P, Wingett SW, Várnai C, Dimond A, Plagnol V, Zerbino D, Schoenfelder S, Javierre B-M, Osborne C, et al. 2016. CHiCAGO: robust detection of DNA looping interactions in Capture Hi-C data. *Genome Biol* **17**: 127.

Chiu TP, Comoglio F, Zhou T, Yang L, Paro R, Rohs R. 2016. DNAshapeR: An R/Bioconductor package for DNA shape prediction and feature encoding. *Bioinformatics* **32**: 1211–1213.

Comoglio F, Paro R. 2014. Combinatorial Modeling of Chromatin Features Quantitatively Predicts DNA Replication Timing in Drosophila. *PLoS Comput Biol* **10**: e1003419.

Comoglio F, Schlumpf T, Schmid V, Rohs R, Beisel C, Paro R. 2015. High-Resolution Profiling of Drosophila Replication Start Sites Reveals a DNA Shape and Chromatin Signature of Metazoan Origins. *Cell Rep* **11**: 821–834.

Diaferia GR, Balestrieri C, Prosperini E, Nicoli P, Spaggiari P, Zerbi A, Natoli G. 2016. Dissection of transcriptional and cis-regulatory control of differentiation in

human pancreatic cancer. *EMBO J* **35**: 1–23.

Dixon JR, Selvaraj S, Yue F, Kim A, Li Y, Shen Y, Hu M, Liu JS, Ren B. 2012. Topological domains in mammalian genomes identified by analysis of chromatin interactions. *Nature* **485**: 376–380.

Dobin A, Davis CA, Schlesinger F, Drenkow J, Zaleski C, Jha S, Batut P, Chaisson M, Gingeras TR. 2013. STAR: ultrafast universal RNA-seq aligner. *Bioinformatics* **29**: 15–21.

Friedman J, Hastie T, Tibshirani R. 2010. Regularization Paths for Generalized Linear Models via Coordinate Descent. *J Stat Softw* **33**.

Grant CE, Bailey TL, Noble WS. 2011. FIMO: Scanning for occurrences of a given motif. *Bioinformatics* **27**: 1017–1018.

Harmston N, Ing-Simmons E, Perry M, Barešić A, Lenhard B. 2015. GenomicInteractions: An R/Bioconductor package for manipulating and investigating chromatin interaction data. *BMC Genomics* **16**: 963.

Heinz S, Benner C, Spann N, Bertolino E, Lin YC, Laslo P, Cheng JX, Murre C, Singh H, Glass CK. 2010. Simple Combinations of Lineage-Determining Transcription Factors Prime cis-Regulatory Elements Required for Macrophage and B Cell Identities. *Mol Cell* **38**: 576–589.

Huber W, Carey VJ, Gentleman R, Anders S, Carlson M, Carvalho BS, Bravo HC, Davis S, Gatto L, Girke T, et al. 2015. Orchestrating high-throughput genomic analysis with Bioconductor. *Nat Methods* **12**: 115–121.

Ignatiadis N, Klaus B, Zaugg JB, Huber W. 2016. Data-driven hypothesis weighting increases detection power in genome-scale multiple testing. *Nat Methods* **13**: 577–580.

Imakaev M, Fudenberg G, McCord RP, Naumova N, Goloborodko A, Lajoie BR, Dekker J, Mirny LA. 2012. Iterative correction of Hi-C data reveals hallmarks of chromosome organization. *Nat Methods* **9**: 999–1003.

Langmead B, Salzberg SL. 2012. Fast gapped-read alignment with Bowtie 2. *Nat Methods* **9**: 357–9.

Lin YC, Benner C, Mansson R, Heinz S, Miyazaki K, Miyazaki M, Chandra V, Bossen C, Glass CK, Murre C. 2012. Global changes in the nuclear positioning of genes and intra- and interdomain genomic interactions that orchestrate B cell fate. *Nat Immunol* **13**: 1196–1204.

Love MI, Huber W, Anders S. 2014. Moderated estimation of fold change and

dispersion for RNA-seq data with DESeq2. *Genome Biol* **15**: 550.

Lun ATL, Chen Y, Smyth GK. 2016. It's DE-licious: A Recipe for Differential Expression Analyses of RNA-seq Experiments Using Quasi-Likelihood Methods in edgeR. In *Statistical Genomics: Methods and Protocols, Methods in Molecular Biology*, Vol. 1418 of http://link.springer.com/10.1007/978-1-4939-3578-9.

Lun ATL, Smyth GK. 2015. Csaw: A Bioconductor package for differential binding analysis of ChIP-seq data using sliding windows. *Nucleic Acids Res* **44**.

Quinlan AR, Hall IM. 2010. BEDTools: A flexible suite of utilities for comparing genomic features. *Bioinformatics* **26**: 841–842.

Schoenfelder S, Furlan-Magaril M, Mifsud B, Tavares-Cadete F, Sugar R, Javierre BM, Nagano T, Katsman Y, Sakthidevi M, Wingett SW, et al. 2015. The pluripotent regulatory circuitry connecting promoters to their long-range interacting elements. *Genome Res* **25**: 582–597.

Servant N, Varoquaux N, Lajoie BR, Viara E, Chen C-J, Vert J-P, Heard E, Dekker J, Barillot E. 2015. HiC-Pro: an optimized and flexible pipeline for Hi-C data processing. *Genome Biol* **16**: 259.

Sing T, Sander O, Beerenwinkel N, Lengauer T. 2005. ROCR : visualizing classifier performance in R. *Bioinformatics* **21**: 3940–3941.

Tarasov A, Vilella AJ, Cuppen E, Nijman IJ, Prins P. 2015. Sambamba: Fast processing of NGS alignment formats. *Bioinformatics* **31**: 2032–2034.

Tibshirani R. 1996. Regression selection and shrinkage via the lasso. *J R Stat Soc B* **58**: 267–288.

Whyte WA, Orlando DA, Hnisz D, Abraham BJ, Lin CY, Kagey MH, Rahl PB, Lee TI, Young RA. 2013. Master Transcription Factors and Mediator Establish Super-Enhancers at Key Cell Identity Genes. *Cell* **153**: 307–319.

Wilson NK, Foster SD, Wang X, Knezevic K, Schütte J, Kaimakis P, Chilarska PM, Kinston S, Ouwehand WH, Dzierzak E, et al. 2010. Combinatorial transcriptional control in blood stem/progenitor cells: Genome-wide analysis of ten major transcriptional regulators. *Cell Stem Cell* **7**: 532–544.

Wilson NK, Schoenfelder S, Hannah R, Sanchez Castillo M, Schutte J, Ladopoulos V, Mitchelmore J, Goode DK, Calero-Nieto FJ, Moignard V, et al. 2016. Integrated genome-scale analysis of the transcriptional regulatory landscape in a blood stem/progenitor cell model. *Blood* **127**: e12–e23.

Yu G, Wang LG, He QY. 2015. ChIP seeker: An R/Bioconductor package for ChIP

peak annotation, comparison and visualization. *Bioinformatics* **31**: 2382–2383.
Zhang Y, Liu T, Meyer CA, Eeckhoute J, Johnson DS, Bernstein BE, Nusbaum C, Myers RM, Brown M, Li W, et al. 2008. Model-based Analysis of ChIP-Seq (MACS). *Genome Biol* **9**: R137.
