# Supplementary material for: Thrombopoietin signaling to chromatin elicits rapid and pervasive epigenome remodeling within poised chromatin architectures
Source: Genome Res. 2018 Mar;28(3):295–309. doi: 10.1101/gr.227272.117 (PMC5848609; doi:10.1101/gr.227272.117)
Supplement: Supplemental Material [file supp_gr.227272.117_Supplemental_Table_S1.pdf]

Supplemental Table S1. ChIP-seq binding profiles in HPC-7 cells used for statistical learning. SS, serum-starved; SR, self-renewing.

**Feature**	**Condition**	**Reference**	**Repository**	**Accession number**
CTCF	SS	This study	GEO	GSE100835
CTCF	TPO 30’	This study	GEO	GSE100835
STAT1 (uSTAT1)	SS	This study	GEO	GSE100835
STAT1 (pSTAT1)	TPO 30’	This study	GEO	GSE100835
CBFA2T3	SR	(Wilson et al. 2016)	ArrayExpress	E-MTAB-3954
CEBPB	SR	(Wilson et al. 2016)	ArrayExpress	E-MTAB-3954
E2F4	SR	(Wilson et al. 2016)	ArrayExpress	E-MTAB-3954
EGR1	SR	(Wilson et al. 2016)	ArrayExpress	E-MTAB-3954
ELF1	SR	(Wilson et al. 2016)	ArrayExpress	E-MTAB-3954
ERG	SR	(Wilson et al. 2010)	GEO	GSE22178
FLI1	SR	(Wilson et al. 2010)	GEO	GSM552233
FOS	SR	(Wilson et al. 2016)	ArrayExpress	E-MTAB-3954
GATA2	SR	(Wilson et al. 2010)	ArrayExpress	GSM552234
GFI1B	SR	(Wilson et al. 2010)	GEO	GSM552235
JUN	SR	(Wilson et al. 2016)	ArrayExpress	E-MTAB-3954
LDB1	SR	(Wilson et al. 2016)	ArrayExpress	E-MTAB-3954
LMO2	SR	(Wilson et al. 2010)	GEO	GSM552237
LYL1	SR	(Wilson et al. 2010)	GEO	GSM552238
MAX	SR	(Wilson et al. 2016)	ArrayExpress	E-MTAB-3954
MEIS1	SR	(Wilson et al. 2010)	GEO	GSM552239
MYB	SR	(Wilson et al. 2016)	ArrayExpress	E-MTAB-3954
MYC	SR	(Wilson et al. 2016)	ArrayExpress	E-MTAB-3954
NFE2	SR	(Wilson et al. 2016)	ArrayExpress	E-MTAB-3954
RUNX1	SR	(Wilson et al. 2010)	GEO	GSM552241
SPI1	SR	(Wilson et al. 2010)	GEO	GSM552240
STAT5 (uSTAT5)	SS	(Park et al. 2015)	GEO	GSE70697
STAT5 (pSTAT5)	TPO 30’	(Park et al. 2015)	GEO	GSE70697

TAL1	SR	(Wilson et al. 2010)	GEO	GSM552242
TCF3	SR	(Calero-nieto et al. 2014)	GEO	GSM1167573

References

Calero-nieto FJ, Ng FS, Wilson NK, Hannah R, Moignard V, Leal-cervantes AI, Jimenez-madrid I, Diamanti E, Wernisch L. 2014. Key regulators control distinct transcriptional programmes in blood progenitor and mast cells. *EMBO J* **33**: 1212-1226.

Park HJ, Li J, Hannah R, Biddie S, Leal-cervantes AI, Flores D, Cruz S, Sexl V, Göttgens B, Green AR. 2015. Cytokine-induced megakaryocytic differentiation is regulated by genome-wide loss of a uSTAT transcriptional program. *EMBO J* **35**: 580–594.

Wilson NK, Foster SD, Wang X, Knezevic K, Schütte J, Kaimakis P, Chilarska PM, Kinston S, Ouwehand WH, Dzierzak E, et al. 2010. Combinatorial transcriptional control in blood stem/progenitor cells: genome-wide analysis of ten major transcriptional regulators. *Cell Stem Cell* **7**: 532–544.

Wilson NK, Schoenfelder S, Hannah R, Sanchez Castillo M, Schutte J, Ladopoulos V, Mitchelmore J, Goode DK, Calero-Nieto FJ, Moignard V, et al. 2016. Integrated genome-scale analysis of the transcriptional regulatory landscape in a blood stem/progenitor cell model. *Blood* **127**: e12–e23.
