# Supplementary material for: Thrombopoietin signaling to chromatin elicits rapid and pervasive epigenome remodeling within poised chromatin architectures
Source: Genome Res. 2018 Mar;28(3):295–309. doi: 10.1101/gr.227272.117 (PMC5848609; doi:10.1101/gr.227272.117)
Supplement: Supplemental Material [file supp_gr.227272.117_Supplemental_Table_S2.pdf]

Supplemental Table S2. Primer sequences used in this study.

**Name**	**Sequence**	**Application**
Calr R1 HindIII	TCATGAGTTCCCCACATCTTTG	Hi-C library QC
Calr R3 HindIII	CTGTGGGCACCAGATGTGTAAAT	Hi-C library QC
Gapdh (Belton et al. 2012)	TATCAAGGGTGCCCGTCACCTTCAGC	Hi-C library QC
Gapdh (Belton et al. 2012)	GGGCTTTTATAGCACGGTTATAAAGT	Hi-C library QC
Hoxa7 fwd HindIII	GGAGGAGGGAAAAGGAGTGATT	Hi-C library QC
Hoxa13 rev HindIII	CAGGCATTATTTGCTGAGAACG	Hi-C library QC
Hist1h2ae fwd HindIII	GGGTAATGGTGTCACTAACTGG	Hi-C library QC
Hist1h3e fwd HindIII	GGGTTTGATGAGTTGGTGAAG	Hi-C library QC
Hist1h4i fwd HindIII	TTGGGCCAAAGCCTATATGA	Hi-C library QC
Hlf fwd	GCGCAGAAAGAACAACATGG	RT-qPCR
Hlf rev	GCCCAGCTCCTTCCTTAAAT	RT-qPCR
Sox4 fwd	AAGGACAGCGACAAGATTCC	RT-qPCR
Sox4 rev	GCCCGACTTCACCTTCTTT	RT-qPCR
Cxcr4 fwd	GTGGATGGTGGTGTTTCAATTC	RT-qPCR
Cxcr4 rev	CTTGGAGTGTGACAGCTTAGAG	RT-qPCR
Myc fwd	CGATTCCACGGCCTTCTC	RT-qPCR
Myc rev	TCTTCCTCATCTTCTTGCTCTTC	RT-qPCR
Gapdh fwd	CATGGCCTTCCGTGTTCCTA	RT-qPCR
Gapdh rev	CCTGCTTCACCACCTTCTTGAT	RT-qPCR
Hlf promoter fwd	GAAGAGAGCACCGGGAAAG	ChIP-qPCR
Hlf promoter rev	CAGACCCGCAATTCACAAAG	ChIP-qPCR
Hlf enhancer fwd	GGCAGAGGCAATCTCTTCAT	ChIP-qPCR
Hlf enhancer rev	GGACTCGTGACATCGTAGTAATG	ChIP-qPCR
Cxcr4 promoter fwd	GGGCAAACAGAAGTCCAAGA	ChIP-qPCR
Cxcr4 promoter rev	CTCAAGTTCTCCACCCGTAAAC	ChIP-qPCR
Spred1 enhancer fwd	GCAGAAAGTGGAAGTGTGAAAG	ChIP-qPCR
Spred1 enhancer rev	GGCAGGAAGCTGGTATCATAAA	ChIP-qPCR
Hlf enhancer uSTAT5+ fwd	GGAGAGACAAAGGTCCCATAAG	ChIP-qPCR
Hlf enhancer uSTAT5+ rev	GCCTGGTATTCAGGGTAACAA	ChIP-qPCR
Cxcr4 enhancer uSTAT5+ fwd	GGGCATCTAGCCAAGCATATC	ChIP-qPCR
Cxcr4 enhancer uSTAT5+ rev	ACTAGAGTCGGAGGCTTTCA	ChIP-qPCR
Sox4-1 uSTAT5+ fwd	TAGCAGGACTCCCAAGAAGA	ChIP-qPCR

Sox4-1 uSTAT5+ rev	CCGCAGTAACACACACCTAA	ChIP-qPCR
Sox4-2 uSTAT5+ fwd	CTGCTGCCGCTCTTAATACA	ChIP-qPCR
Sox4-2 uSTAT5+ rev	CACTTGAGAGGGAGCAAATCA	ChIP-qPCR

References

Belton J, Mccord RP, Gibcus JH, Naumova N, Zhan Y, Dekker J. 2012. Hi-C : A comprehensive technique to capture the conformation of genomes. *EMBO J* **58**: 268–276.
